# Arterio-venous gradient of active interleukin-18 is associated with diastolic dysfunction: a cross-sectional study

**DOI:** 10.1093/eschf/xvaf041

**Published:** 2026-01-19

**Authors:** Hirofumi Makino, Yu Yasuda, Yusuke Morita, Hiroshi Kawahara, Yuzo Kagawa, Akihiro Endo, Hiroki Kamino, Kazuaki Tanabe, Takeshi Urano, Keizo Kanasaki

**Affiliations:** Internal Medicine 1, Shimane University Faculty of Medicine, 89-1 Enya-cho, Izumo, Shimane 693-8501, Japan; Internal Medicine 4, Shimane University Faculty of Medicine, Izumo 693-8501, Japan; Internal Medicine 4, Shimane University Faculty of Medicine, Izumo 693-8501, Japan; Internal Medicine 4, Shimane University Faculty of Medicine, Izumo 693-8501, Japan; Internal Medicine 4, Shimane University Faculty of Medicine, Izumo 693-8501, Japan; Internal Medicine 4, Shimane University Faculty of Medicine, Izumo 693-8501, Japan; Center for Vaccines and Therapeutic Antibodies for Emerging Infectious Diseases, Shimane University, Izumo 693-8501, Japan; Internal Medicine 4, Shimane University Faculty of Medicine, Izumo 693-8501, Japan; Center for Vaccines and Therapeutic Antibodies for Emerging Infectious Diseases, Shimane University, Izumo 693-8501, Japan; Internal Medicine 1, Shimane University Faculty of Medicine, 89-1 Enya-cho, Izumo, Shimane 693-8501, Japan; Center for Integrated Kidney Research and Advance, Shimane University Faculty of Medicine, Izumo 693-8501, Japan

**Keywords:** Heart failure, Diastolic dysfunction, Interleukin-18, Inflammatory cytokines, Cardiac fibrosis

## Abstract

**Introduction:**

Diastolic dysfunction is a key determinant of symptoms and prognosis in heart failure (HF). Interleukin (IL)-18 and IL-6 are key inflammatory cytokines in HF; however, their local activation within the cardiopulmonary circulation and relevance to diastolic dysfunction remain unclear. This exploratory study investigated associations between arterio-venous (A/V) cytokine gradients and diastolic dysfunction.

**Methods:**

Eighty-seven patients undergoing diagnostic cardiac catheterization were enrolled. Paired arterial samples from the left ventricle (LV) or ascending aorta and peripheral venous samples were obtained simultaneously or within 24 h of the procedure. Active IL-18 (aIL-18) and IL-6 concentrations were measured, and associations with echocardiographic and clinical parameters were evaluated. Active interleukin-18-induced fibrotic responses were evaluated in human cardiac fibroblasts.

**Results:**

The cohort exhibited impaired myocardial relaxation (septal *e*′: 5.3 ± 2.0 cm/s) and preserved ejection fraction (57.4 ±11.8%). The aIL-18 A/V ratio correlated with average *E*/*e*′ (*r* = 0.31, *P* < .01), tricuspid regurgitation pressure gradient (*r* = 0.29, *P* = .015), and Heart Failure Association-Pre-test assessment, Echocardiography & natriuretic peptide, Functional testing, Final aetiology (HFA-PEFF) score (*r* = 0.23, *P* = .034). Correlations between the aIL-18 A/V ratio and *E*/*e*′ were more pronounced in non-diabetic patients and in those with elevated LV filling pressure (average *E*/*e*′ ≥ 15). Interleukin-6 correlated with albuminuria and pulmonary function; however, no synergistic interaction with aIL-18 was observed. *In vitro*, aIL-18 stimulated fibroblast proliferation and collagen synthesis.

**Conclusion:**

The aIL-18 A/V ratio correlated with markers of diastolic dysfunction, particularly in patients with increased filling pressure. These exploratory findings indicate an association between local IL-18 gradients and diastolic dysfunction, warranting further investigation.

## Graphical Abstract

**Graphical Abstract:**
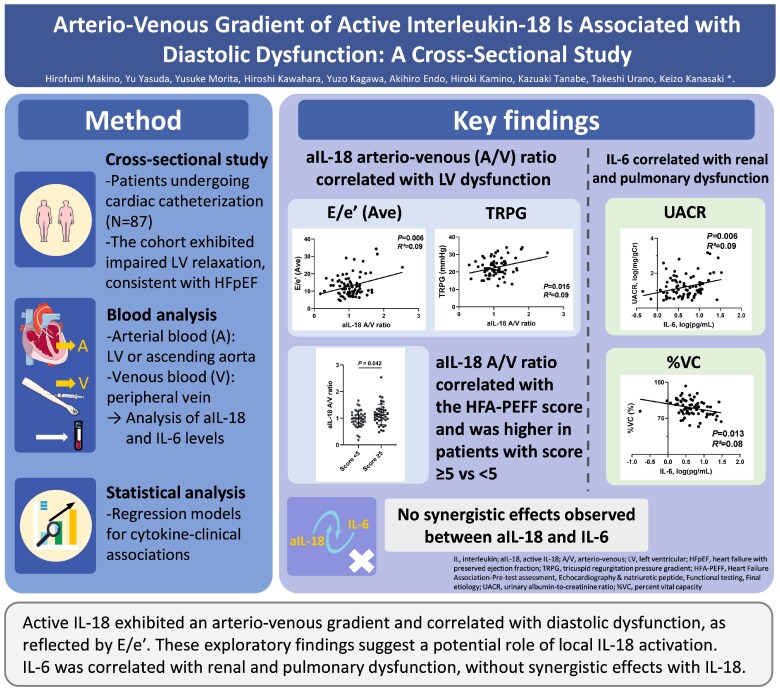
Graphical abstract summarizing the study design and key findings, showing that an increased arterio-venous gradient of active interleukin-18 is associated with left ventricular diastolic dysfunction, whereas interleukin-6 is related to renal and pulmonary dysfunction without synergistic effects.

## Introduction

Diastolic dysfunction is frequently encountered during cardiovascular evaluation and is an important determinant of symptoms and adverse outcomes in individuals with heart failure (HF), where it has been associated with increased risks of cardiovascular events and mortality.^1^ It represents a common pathophysiological feature across HF phenotypes, irrespective of ejection fraction.^2,3^ Its prevalence continues to increase alongside population ageing and the rising burden of cardiometabolic comorbidities.^4,5^ Diastolic abnormalities are associated with impaired myocardial relaxation, elevated left ventricular (LV) filling pressures, exercise intolerance, and congestion.^6,7^ Despite its clinical importance, the biological pathways underlying diastolic dysfunction remain incompletely understood.

Inflammatory cytokines such as interleukin (IL)-18 and IL-6 are implicated in HF pathogenesis, and elevations in these cytokines have been associated with myocardial fibrosis and diastolic dysfunction.^8,9^ Interleukin-18 is synthesized as an inactive precursor and becomes biologically active through inflammasome-dependent cleavage in response to cellular stress signals.^10,11^ Increased levels of active IL-18 (aIL-18) have been reported in both pressure-overload models and clinical HF.^12,13^ Interleukin-6 is associated with cardiovascular and renal dysfunction and is under investigation as a therapeutic target in HF^14–16^. However, most available evidence comes from peripheral blood sampling, leaving it uncertain whether these cytokines show local gradients within the cardiopulmonary circulation or whether such local behaviour is associated with indices of diastolic dysfunction in HF.

Given these gaps, we conducted an exploratory cross-sectional study in patients undergoing diagnostic cardiac catheterization, using paired arterial samples obtained from the LV or ascending aorta and peripheral venous samples. We investigated whether arterio-venous (A/V) cytokine gradients of aIL-18 and IL-6 were associated with echocardiographic and clinical markers of diastolic dysfunction. In addition, *in vitro* studies using human cardiac fibroblasts (HCFs) were performed to characterize cellular responses to aIL-18.

## Methods

### Study design and participants

This cross-sectional study included 87 patients admitted to Shimane University Hospital between 31 August 2021 and 31 January 2023 who underwent cardiac catheterization. Patients were consecutively enrolled from those undergoing diagnostic cardiac catheterization during the study period. Indications for catheterization included suspected coronary artery disease, evaluation of valvular heart disease, and diagnostic assessment for suspected cardiomyopathies as part of the general work-up for cardiovascular disorders. Eligible participants were aged ≥18 years. We screened 95 patients and excluded 8 on maintenance haemodialysis, leaving 87 in the final cohort. All participants provided written informed consent. This study protocol was approved by the institutional ethics committee (ID: 20201026-1) and conducted in accordance with the Declaration of Helsinki.

### Baseline data collection and clinical definitions

All patients underwent standardized clinical and laboratory assessments at baseline. Demographic and clinical variables included age, sex, and history of hypertension (HT), diabetes mellitus (DM), dyslipidaemia, and smoking. Medication use at catheterization was abstracted from charts [statins, angiotensin-converting enzyme inhibitors/angiotensin receptor blockers (ACEi/ARB), β-blockers, sodium-glucose cotransporter-2 (SGLT2) inhibitors]. Laboratory parameters included markers of glucose metabolism, renal function, urinary albumin, and B-type natriuretic peptide (BNP). Transthoracic echocardiography was performed during the same admission (within 2 weeks), most often 1 day before catheterization (*n* = 36) or 2 days before (*n* = 21), and within 3 days in more than 80% of patients. Left ventricular ejection fraction (LVEF) was measured by the modified Simpson method. Because catheterization was part of routine care, invasive pressure measurements such as LV end-diastolic pressure (LVEDP) were not systematically obtained. Diastolic function was therefore evaluated using echocardiographic surrogates, including average *E*/*e*′. Hypertension, DM, and dyslipidaemia were defined by prior clinical diagnosis and treatment. Heart failure with preserved ejection fraction (HFpEF) was defined as LVEF ≥50% and Heart Failure Association-Pre-test assessment, Echocardiography & natriuretic peptide, Functional testing, Final aetiology (HFA-PEFF) score ≥ 5, according to ESC guidelines. Aortic stenosis (AS) was diagnosed following AHA/ACC criteria.

### Blood sample collection

Arterial and venous blood samples were collected simultaneously during catheterization in 16 patients. In the remaining patients, venous blood was obtained separately, on the same day (*n* = 55), the next day (*n* = 11), or 2 days later (*n* = 3). In most cases, venous sampling preceded catheterization; however, in four cases, arterial sampling was performed before venous sampling. Arterial samples were typically drawn from the LV; in patients with AS, they were collected from the ascending aorta to avoid crossing the stenotic valve. Venous samples were obtained from a peripheral upper-extremity vein. All samples were centrifuged at 1500 *g* for 10 min and stored at −80°C until analysis. Of the 87 patients enrolled, paired arterial and venous samples were available in 85 patients. In two patients, only venous samples were obtained; these patients were included in analyses involving venous concentrations but excluded from analyses of A/V ratios.

### Cytokine measurement

Serum aIL-18 concentrations were measured with a commercial ELISA kit (E-I-002, mAbProtein, Japan) that employs a monoclonal antibody specific to the neoepitope of aIL-18. Interleukin-6 concentrations were measured using a human IL-6 ELISA kit (D6050B, R&D Systems, USA).

### *In vitro* assays

Human cardiac fibroblasts (C-12375, PCI, Heidelberg, Germany) were cultured under standard conditions. After serum starvation in 0.1% fetal bovine serum (FBS)–supplemented Dulbecco’s modified Eagle’s medium (DMEM) for 24 h, cells were treated with aIL-18 (1, 10, or 100 ng/ml) for 24 h. Protein expression of α-smooth muscle actin (α-SMA; 1:3000, ab5694, Abcam, Cambridge, UK) and collagen type I alpha 1 (1:1000, #72026, Cell Signaling Technology, Danvers, MA, USA) was analysed by western blotting, and cell proliferation was assessed with a BrdU ELISA kit (ab126556, Abcam).

### Statistical analysis

Continuous variables are presented as mean ± standard deviation (SD). Arterial and venous cytokine concentrations were log_10_-transformed to approximate normality, whereas A/V ratios were calculated from raw values. Normality was tested with the Shapiro–Wilk test. For non-normally distributed variables, between-group comparisons used the Mann–Whitney *U* test. Relationships between continuous variables were assessed by Pearson correlation and simple linear regression (GraphPad Prism v10.0.2). Comparisons across multiple groups used one-way analysis of variance with Tukey’s *post hoc* test. Multivariable linear regression was performed in SPSS v26 (IBM, USA), adjusting for age, sex, body mass index (BMI), smoking, DM, HT, urinary albumin-to-creatinine ratio (UACR), and estimated glomerular filtration rate (eGFR). Univariable models used all available data; multivariable analyses were restricted to complete cases (*n* = 86, one missing UACR). Two-sided *P*-value <.05 was considered statistically significant. For scatter plots, IL-6 values were displayed after log(*x* + 1) transformation to avoid negative values, and IL-18 values after log transformation. Figures were prepared using GraphPad Prism and Microsoft Excel 2021.

## Results

### Patient characteristics

**Figure 1:**
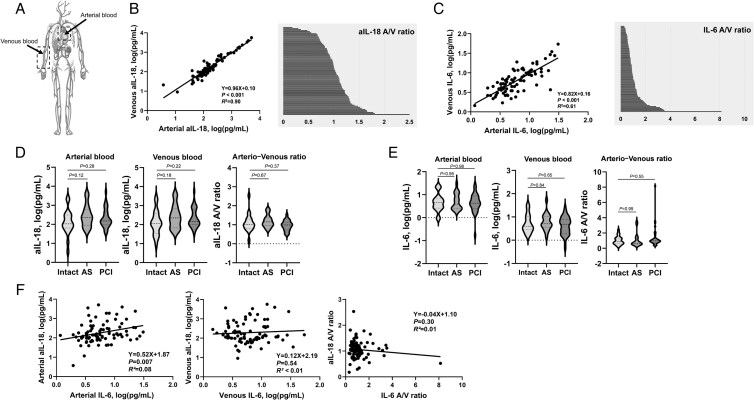
Comparison of arterial and venous cytokine levels and arterio-venous ratios of active interleukin-18 and interleukin-6. (A) Schematic illustration of blood sampling sites. Arterial blood was collected from the left ventricle or ascending aorta (in patients with aortic stenosis), and venous blood was drawn from a peripheral upper-extremity vein. (B) Scatter plot showing the correlation between arterial and venous concentrations of active interleukin-18 and the corresponding histogram displaying the distribution of active interleukin-18 arterio-venous ratios (>1 indicates higher arterial than venous concentrations, <1 indicates the opposite). (C) Scatter plot showing the correlation between arterial and venous concentrations of interleukin-6 and the corresponding histogram displaying the distribution of interleukin-6 arterio-venous ratios. (D and E) Violin plots comparing arterial, venous, and arterio-venous ratio values of active interleukin-18 (D) and interleukin-6 (E) across three clinical subgroups: patients without significant coronary stenosis (Intact), those with aortic stenosis, and those post-percutaneous coronary intervention. (F) Correlation analyses between arterial and venous interleukin-6 and active interleukin-18, as well as their arterio-venous ratios. Log(*x* + 1) transformation was applied in scatter plots to avoid negative values for interleukin-6, whereas interleukin-18 values were log-transformed without additional adjustment. Each scatter plot displays the *P*-value and coefficient of determination (*R*^2^). *P* < .05 was considered statistically significant. Panel A was adapted from Servier Medical Art (https://smart.servier.com), licensed under Creative Commons Attribution 4.0 International (CC BY 4.0)

This study included 87 patients who underwent diagnostic cardiac catheterization. Arterial blood was obtained from the LV or ascending aorta during the procedure. Venous blood was collected from a peripheral upper-extremity vein. In 16 patients, sampling was simultaneous with catheterization, whereas in the others, venous sampling was performed mostly within 24 h before or after the procedure (*Figure 1A*). The most common cardiac conditions were stable angina (32.2%), possible angina (21.8%), and AS (20.7%) (see Supplementary data online, *Table S1*).

Baseline characteristics are summarized in *Table 1*. The cohort was elderly (73.6 ± 9.8 years), and 64% were male. Hypertension and DM were present in 86% and 31% of patients, respectively. Regarding medications, 63% received statins, 54% ACEi/ARB, 30% β-blockers, and 14% SGLT2 inhibitors. Median BNP was 68.3 pg/ml [interquartile range (IQR) 25.9–176.6]. Echocardiography showed preserved systolic function (LVEF: 57.4 ± 11.8%) together with impaired relaxation and elevated LV filling pressure, including reduced septal *e*′ velocity (5.3 ± 2.0 cm/s) and increased average *E*/*e*′ (13.0 ± 6.2). Structural abnormalities included interventricular septal thickening (11.3 ± 2.3 mm) and left atrial enlargement (left atrial volume index 46.7 ± 22.7 ml/m^2^). The HFA-PEFF score,^17^ which integrates functional, structural, and biomarker domains, averaged 4.1 ± 1.8 with a median of 4.5. Reflecting a skewed distribution, nearly half of the cohort scored ≥ 5, meeting the diagnostic threshold for HFpEF.

**Table 1:** Baseline characteristics and clinical profiles

Characteristic	Patient (*N* = 87)
Age, years	73.6 ± 9.8
Men, no. (%)	56 (64.4)
Body weight, kg	59.9 ± 14.1
BMI, kg/m^2^	23.2 ± 3.9
Systolic blood pressure, mmHg	129.5 ± 18.5
Diastolic blood pressure, mmHg	74.5 ± 13.7
Diabetes mellitus, no. (%)	27 (31.0)
Hypertension, no. (%)	75 (86.2)
Dyslipidaemia, no. (%)	63 (72.4)
Smoking history, no. (%)	54 (62.1)
Statins, no. (%)	55 (63.2)
ACEi/ARB, no. (%)	47 (54.0)
β-Blockers, no. (%)	26 (29.9)
SGLT2 inhibitors, no. (%)	12 (13.8)
HFA-PEFF score	4.1 ± 1.8
0–1, no. (%)	6 (6.9)
2–4, no. (%)	37 (42.5)
≥5, no. (%)	44 (50.6)
Blood/urine examination	
FPG, mg/dl	115.1 ± 32.9
HbA1c, %	6.2 ± 0.9
eGFR, ml/min/1.73 m^2^	61.6 ± 20.1
UACR (IQR), mg/gCr	10.9 (5.4–30.7)
BNP (IQR), pg/ml	68.3 (25.9–176.6)
X-ray	
Cardio thoracic ratio, %	51.6 ± 8.8
Echocardiography	
LVEF, %	57.4 ± 11.8
LVDd, mm	44.7 ± 6.9
LVDs, mm	30.9 ± 7.8
IVSth, mm	11.3 ± 2.3
PWth, mm	10.7 ± 1.7
LAD, mm	39.5 ± 7.2
LAVI, ml/m^2^	46.7 ± 22.7
*E*/*A*	0.8 ± 0.4
Septal *e*′ (cm/s)	5.3 ± 2.0
Average *E*/*e*′	13.0 ± 6.2
TRVmax (m/s)	2.3 ± 0.3
TRPG, mmHg	22.4 ± 5.2
Respiratory function test	
FEV_1.0%_	73.7 ± 9.2
%VC, %	96.4 ± 16.6

Data are presented as the mean ± SD or median (25th–75th percentile). Categorical variables are expressed as count (percentage).

BMI, body mass index; ACEi/ARB, angiotensin-converting enzyme inhibitors/angiotensin receptor blockers; SGLT2, sodium-glucose cotransporter-2; HFA-PEFF, Heart Failure Association–Pre-test assessment, Echocardiography & natriuretic peptide, Functional testing, Final aetiology; FPG, fasting plasma glucose; HbA1c, haemoglobin A1c; eGFR, estimated glomerular filtration rate; UACR, urinary albumin-to-creatinine ratio; BNP, brain natriuretic peptide; LVEF, left ventricular ejection fraction; LVDd, left ventricular diastolic dimension; LVDs, left ventricular systolic dimension; IVSth, interventricular septal thickness; PWth, posterior wall thickness; LAD, left atrial dimension; LAVI, left atrial volume index; TRVmax, maximal tricuspid regurgitant velocity; TRPG, tricuspid regurgitant pressure gradient; FEV1.0%, forced expiratory volume in 1 s; %VC, per cent vital capacity.

### Arterio-venous cytokine profiles

For aIL-18, mean arterial and venous concentrations were 445.6 and 488.6 pg/ml; median values were 142.0 (94.8–307.6) and 143.6 (91.9–392.0) pg/ml. The A/V ratio averaged 1.06 [median 1.03 (0.86–1.22)], consistent with a modest gradient overall. A subset of patients (47/87, 54%) showed elevated arterial levels, illustrated by A/V ratios > 1 (*Figure 1B*), which may suggest local activation in the cardiopulmonary circulation. By definition, A/V ratios > 1 indicate higher arterial than venous concentrations, consistent with net *trans*-pulmonary release/activation or differential clearance; A/V ratios < 1 suggest net peripheral release or non-pulmonary handling. The A/V ratio was not significantly associated with obesity, smoking, DM, HT, or renal dysfunction (see Supplementary data online, *Table S2*).

For IL-6, mean arterial and venous concentrations were 6.51 and 7.05 pg/ml; median values were 4.29 (2.47–7.78) and 4.29 (2.47–9.0) pg/ml. The A/V ratio averaged 1.16 [median 0.90 (0.64–1.27)], indicating a more pronounced gradient than for aIL-18 (*Figure 1C*), possibly reflecting greater peripheral production or differences in clearance.

We further compared cytokine levels across three groups: patients who underwent percutaneous coronary intervention (PCI), patients without significant coronary stenosis (Intact), and patients with AS. No significant differences were observed (*Figure 1D* and *E*). Arterial IL-6 correlated positively with arterial aIL-18 (*P* = .007), whereas venous levels and A/V ratios showed no such relationship (*Figure 1F*).

### Arterio-venous gradient of active interleukin-18 and diastolic dysfunction

**Figure 2:**
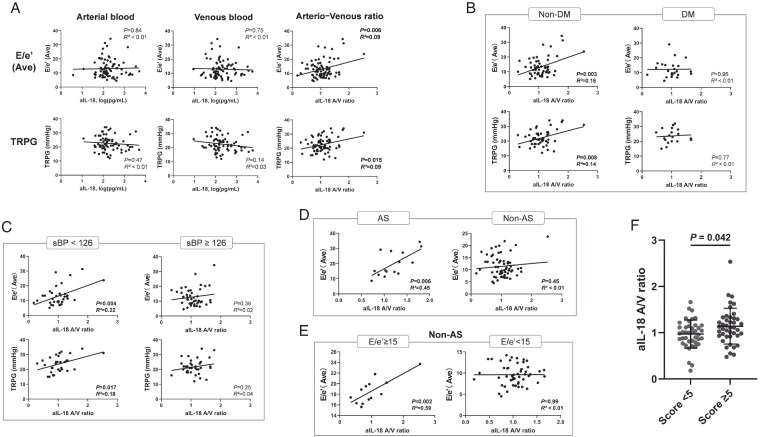
Association between active interleukin-18 levels and clinical parameters of diastolic function. (A) Scatter plots showing correlations between active interleukin-18 levels (arterial, venous, and arterio-venous ratio) and average *E*/*e*′ or tricuspid regurgitation pressure gradient. (B and C) Subgroup analyses stratified by diabetes status (diabetes mellitus vs non-diabetes mellitus) (B) and systolic blood pressure (<126 vs ≥126 mmHg) (C). (D) Association between active interleukin-18 arterio-venous ratio and average *E*/*e*′ in patients with aortic stenosis and those without aortic stenosis. (E) Correlation between active interleukin-18 arterio-venous ratio and *E*/*e*′ in non-aortic stenosis patients, further stratified by *E*/*e*′ ≥15 or <15. (F) Dot plot comparing active interleukin-18 arterio-venous ratio between patients with Heart Failure Association-PEFF score <5 and those meeting the diagnostic threshold for heart failure with preserved ejection fraction (≥5). Plots show individual values with error bars indicating mean ± standard deviation. Statistical comparison was performed using the Mann–Whitney *U* test. *P* < .05 was considered statistically significant. (A–E) *R*^2^ values

To examine associations between aIL-18 and clinical parameters, we performed Pearson correlation and simple linear regression analyses (*Table 2*). Arterial aIL-18 levels correlated inversely with body weight (*r* = −0.25, *P* = .021) but not with metabolic, renal, or respiratory indices. Absolute arterial or venous levels were not related to cardiac function, whereas the aIL-18 A/V ratio correlated positively with average *E*/*e*′ (*r* = 0.31, *P* < .01) and tricuspid regurgitation pressure gradient (TRPG, *r* = 0.29, *P* = .015) (*Figure 2A*). B-type natriuretic peptide showed a positive trend (*r* = 0.21, *P* = .054) but was not significant.

**Table 2:** Regression and correlation analysis of active interleukin-18 with clinical parameters

	Arterial (A)			Venous (V)			Arterio-venous (A/V)		
	*r*	*β* (95% CI)	*P*-value	*r*	*β* (95% CI)	*P*-value	*r*	*β* (95% CI)	*P*-value
Age, years	0.06	1.08 (−2.90, 5.07)	.59	−0.04	−0.64 (−4.54, 3.26)	.75	0.17	4.81 (−1.19, 10.8)	.11
Height, cm^2^	−0.17	−3.64 (−8.34, 1.07)	.13	−0.13	−2.81 (−7.38, 1.76)	.22	−0.07	−2.33 (−9.59, 4.93)	.53
BW, kg	**−0**.**25**	−6.70 (−12.4, −1.03)	.021*	**−0**.**21**	−5.54 (−11.0, −0.07)	.047*	−0.06	−2.51 (−11.4, 6.35)	.57
BMI, kg/m^2^	−0.18	−1.36 (−2.94, 0.22)	.09	−0.16	−1.15 (−2.65, 0.35)	.13	−0.02	−0.25 (−2.66, 2.16)	.84
sBP, mmHg	0.04	1.31 (−6.28, 8.89)	.73	−0.02	−0.50 (−7.80, 6.79)	.89	0.04	2.06 (−9.39, 13.5)	.72
dBP, mmHg	−0.01	−0.23 (−5.50, 5.34)	.94	−0.03	−0.72 (−6.15, 4.70)	.79	0.03	1.31 (−7.14, 9.77)	.76
SpO_2_, %	−0.05	−0.13 (−0.65, 0.94)	.63	−0.08	−0.19 (−0.69, 0.31)	.45	0.09	0.33 (−0.464, 1.12)	.41
FPG, mg/dl	−0.04	−2.23 (−15.8, 11.3)	.74	0.05	2.74 (−10.4, 15.9)	.68	−0.18	−16.8 (−37.1, 3.60)	.11
HbA1c, %	−0.13	−0.21 (−0.57, 0.14)	.24	−0.10	−0.15 (−0.49, 0.19)	.38	−0.11	−0.27 (−0.81, 0.27)	.32
UACR, g/gCr	0.06	0.07 (−0.18, 0.32)	.58	0.09	0.10 (−0.15, 0.35)	.41	0.12	0.21 (−0.18, 0.59)	.28
eGFR, ml/min/1.73 m^2^	0.10	0.03 (−0.03, 0.08)	.34	−0.01	−0.002 (−0.06, 0.05)	.95	−0.08	−0.03 (−0.11, 0.05)	.46
BNP, pg/ml	0.14	0.16 (−0.08, 0.39)	.19	0.09	0.09 (−0.13, 0.32)	.41	0.21	0.35 (−0.006, 0.70)	.054
CTR, %	0.08	1.38 (−2.25, 5.01)	.45	0.04	0.63 (−2.86, 4.12)	.72	0.11	2.90 (−2.62, 8.42)	.30
LVEF, %	−0.02	−0.38 (−5.20, 4.45)	.88	−0.02	−0.39 (−5.12, 4.34)	.87	−0.18	−5.77 (−12.9, 1.37)	.11
LVDd, mm	−0.08	−1.03 (−3.93, 1.86)	.48	−0.12	−1.50 (−4.26, 1.26)	.28	0.18	3.63 (−0.66, 7.92)	.096
LVDs, mm	−0.08	−1.13 (−4.35, 2.09)	.49	−0.10	−1.36 (−4.47, 1.75)	.39	0.21	4.64 (−0.10, 9.39)	.055
IVSth, mm	0.04	0.17 (−0.76, 1.09)	.72	0.10	0.41 (−0.48, 1.31)	.36	0.07	0.46 (−0.92, 1.84)	.51
PWth, mm	0.02	0.07 (−0.63, 0.77)	.84	0.08	0.24 (−0.44, 0.92)	.48	0.06	0.30 (−0.75, 1.35)	.57
LAD, mm	−0.12	−1.64 (−4.64, 1.36)	.28	−0.17	−2.29 (−5.14, 0.57)	.11	0.11	2.28 (−2.25, 6.81)	.32
LAVI, ml/m^2^	−0.05	−2.19 (−12.5, 8.15)	.67	−0.07	−3.00 (−12.4, 6.39)	.53	0.11	7.50 (−7.40, 22.4)	.32
*E*/*A*	0.19	0.13 (−0.03, 0.29)	.10	0.11	0.08 (−0.08, 0.23)	.33	0.12	0.13 (−0.12, 0.38)	.31
Septal *e*′, cm/s	−0.05	−0.19 (−1.05, 0.67)	.66	−0.06	−0.21 (−1.02, 0.61)	.62	−0.13	−0.72 (−1.94, 0.51)	.25
Ave *E*/*e*′	0.02	0.28 (−2.43, 2.99)	.84	−0.04	−0.42 (−2.99, 2.16)	.75	**0**.**31**	5.27 (1.57, 8.98)	.006**
TRVmax, m/s	−0.10	−0.05 (−0.18, 0.08)	.42	−0.18	−0.09 (−0.21, 0.03)	.14	**0**.**26**	0.20 (0.02, 0.38)	.031*
TRPG, mmHg	−0.09	−0.90 (−3.38, 1.59)	.47	−0.18	−1.70 (−3.96, 0.56)	.14	**0**.**29**	4.24 (0.86, 7.62)	.015*
HFA-PEFF score	−0.02	−0.06 (−0.82, 0.71)	.89	−0.04	−0.12 (−0.85, 0.61)	.74	**0**.**23**	1.17 (0.09, 2.26)	.034*
FEV_1.0%_, %	−0.08	−1.38 (−5.68, 2.92)	.52	−0.07	−1.11 (−5.13, 2.90)	.58	0.13	3.40 (−2.82, 9.61)	.28
%VC, %	−0.09	−3.02 (−10.8, 4.75)	.44	−0.07	−2.20 (−9.46, 5.06)	.55	−0.20	−9.54 (−20.7, 1.58)	.091

Simple regression analysis of A, V, and A/V ratio of aIL-18 with clinical parameters.

aIL-18, UACR, eGFR, and BNP were log-transformed to approximate a normal distribution. A *P*-value < .05 was considered statistically significant. *P < .05; **P < .01. Bold values indicate statistical significance. Pearson correlation coefficients (*r*), regression coefficients (*β*), 95% CIs, and *P*-values are presented.

aIL-18, active interleukin-18; BW, body weight; BMI, body mass index; sBP, systolic blood pressure; dBP, diastolic blood pressure; SpO_2_, peripheral capillary oxygen saturation; FPG, fasting plasma glucose; HbA1c, haemoglobin A1c; UACR, urinary albumin-to-creatinine ratio; eGFR, estimated glomerular filtration rate; BNP, B-type natriuretic peptide; CTR, cardiothoracic ratio; LVEF, left ventricular ejection fraction; LVDd, left ventricular diastolic dimension; LVDs, left ventricular systolic dimension; IVSth, interventricular septal thickness; PWth, posterior wall thickness; LAD, left atrial dimension; LAVI, left atrial volume index; TRVmax, maximal tricuspid regurgitant velocity; TRPG, tricuspid regurgitant pressure gradient; HFA-PEFF, Heart Failure Association-Pre-test assessment, Echocardiography and natriuretic peptide, Functional testing, Final aetiology; FEV1.0%, forced expiratory volume in 1 s; %VC, percent vital capacity.

Subgroup analyses demonstrated significant associations between the aIL-18 A/V ratio and *E*/*e*′ in patients without DM and in those with lower systolic blood pressure (sBP), defined as below the cohort median of 126 mmHg (*Figure 2B* and *C*). To further evaluate how the IL-18 A/V ratio relates to diastolic indices, we examined subgroups defined by cardiac loading status. Strong correlations were also observed in patients with AS (*r* = 0.67, *P* < .01) and in non-AS patients with elevated LV filling pressure (*E*/*e*′ ≥ 15; *r* = 0.77, *P* < .01) (*Figure 2D* and *E*), suggesting stronger associations with diastolic indices in these haemodynamic subgroups. The aIL-18 A/V ratio also correlated with the HFA-PEFF score (*r* = 0.23, *P* = .034) and was higher in patients with a score ≥ 5 than in those with <5 (*Figure 2F*). In regression analyses, this association showed only a non-significant trend after multivariable adjustment (*P* = .076) and was further attenuated when renal indices were included (*P* = .13; Supplementary data online, *Table S3*).

In multivariable models (*Table 3*), the aIL-18 A/V ratio was consistently and independently associated with average *E*/*e*′ across all four models. For TRPG, the association was significant in Models 1–3, but lost statistical significance after additional adjustment for renal function (Model 4, *P* = .073). Among covariates, BMI and smoking were positively associated with *E*/*e*′ but not with TRPG. The associations of the aIL-18 A/V ratio with both indices remained significant even after adjustment for these factors (Model 3). Age showed the strongest positive association with *E*/*e*′, whereas male sex was inversely associated. These results are consistent with the known age-related decline in diastolic function and potential sex-related remodelling differences. In exploratory analyses, medication use was not associated with the IL-18 A/V ratio (see Supplementary data online, *Table S4*). Angiotensin-converting enzyme inhibitors/angiotensin receptor blockers use correlated with higher *E*/*e*′ and TRPG; however, the associations of the aIL-18 A/V ratio with these indices remained significant after additional adjustment for ACEi/ARB (see Supplementary data online, *Table S5*).

**Table 3:** Multivariable linear regression analysis of active interleukin-18 arterio-venous ratio with average *E*/*e*′ and tricuspid regurgitation pressure gradient

Ave *E*/*e*′					TRPG				
	Model 1 (unadjusted)	Model 2 (+age, sex)	Model 3 (+BMI, smoking, DM, HT)	Model 4 (+UACR, eGFR)		Model 1 (unadjusted)	Model 2 (+age, sex)	Model 3 (+BMI, smoking, DM, HT)	Model 4 (+UACR, eGFR)
Intercept	*B* = 7.55 ***P*** **=** .001** (95% CI: 3.41, 11.69)	*B* = −5.02 *P* = .33 (95% CI: −15.20, 5.16)	*B* = −22.5 ***P*** **=** .002** (95% CI: −36.3, −8.76)	*B* = −9.50 *P* = .47 (95% CI: −35.5, 16.6)	**Intercept**	*B* = 18.1 ***P*** **<** .001*** (95% CI: 14.2, 22.0)	*B* = 4.30 *P* = .45 (95% CI: −6.97, 15.6)	*B* = 5.00 *P* = .52 (95% CI: −10.6, 20.6)	*B* = 1.61 *P* = .91 (95% CI: −27.5, 30.7)
aIL-18 A/V ratio	*B* = 5.10 ***P*** **=** .008** (95% CI: 1.39, 8.81)	*B* = 4.24 ***P*** **=** .015* (95% CI: 0.86, 7.63)	*B* = 4.80 ***P*** **=** .004** (95% CI: 1.63, 7.96)	*B* = 4.79 ***P*** **=** .004** (95% CI: 1.58, 8.00)	**aIL-18 A/V ratio**	*B* = 4.20 ***P*** **=** .017* (95% CI: 0.78, 7.62)	*B* = 3.64 ***P*** **=** .031* (95% CI: 0.34, 6.93)	*B* = 3.90 ***P*** **=** .022* (95% CI: 0.58, 7.22)	*B* = 3.07 *P* = .073 (95% CI: −0.30, 6.44)
Age		*B* = 0.21 ***P*** **=** .002** (95% CI: 0.08, 0.34)	*B* = 0.30 ***P*** **<** .001*** (95% CI: 0.17, 0.42)	*B* = 0.28 ***P*** **<** .001*** (95% CI: 0.14, 0.41)	**Age**		*B* = 0.20 ***P*** **=** .007** (95% CI: 0.06, 0.35)	*B* = 0.21 ***P*** **=** .006** (95% CI: 0.06, 0.36)	*B* = 0.19 ***P*** **=** .016* (95% CI: 0.04, 0.34)
Sex		*B* = −2.98 ***P*** **=** .029* (95% CI: −5.65, −0.31)	*B* = −6.76 ***P*** **<** .001*** (95% CI: −10.2, −3.31)	*B* = −6.48 ***P*** **=** .001** (95% CI: −10.1, −2.91)	**Sex**		*B* = −0.95 *P* = .46 (95% CI: −3.46, 1.57)	*B* = −0.74 *P* = .66 (95% CI: −4.14, 2.65)	*B* = −0.25 *P* = .89 (95% CI: −3.72, 3.23)
BMI			*B* = 0.46 ***P*** **=** .004** (95% CI: 0.15, 0.76)	*B* = 0.48 ***P*** **=** .003** (95% CI: 0.17, 0.78)	**BMI**			*B* = 0.02 *P* = .92 (95% CI: −0.39, 0.43)	*B* = 0.08 *P* = .70 (95% CI: −0.33, 0.48)
Smoking			*B* = 5.11 ***P*** **=** .004** (95% CI: 1.71, 8.51)	*B* = 4.87 ***P*** **=** .007** (95% CI: 1.35, 8.39)	**Smoking**			*B* = −0.70 *P* = .68 (95% CI: −4.04, 2.65)	*B* = −1.38 *P* = .42 (95% CI: −4.79, 2.04)
DM			*B* = 0.08 *P* = .95 (95% CI: −2.40, 2.56)	*B* = 0.35 *P* = .78 (95% CI: −2.19, 2.89)	**DM**			*B* = 2.29 *P* = .090 (95% CI: −0.37, 4.94)	*B* = 1.99 *P* = .14 (95% CI: −0.65, 4.63)
HT			*B* = −0.61 *P* = .71 (95% CI: −3.89, 2.67)	*B* = −1.09 *P* = .53 (95% CI: −4.55, 2.37)	**HT**			*B* = −2.86 *P* = .11 (95% CI: −6.40, 0.68)	*B* = −3.34 *P* = .072 (95% CI: −6.99, 0.31)
UACR				*B* = −0.57 *P* = .58 (95% CI: −2.59, 1.46)	**UACR**				*B* = 2.30 ***P*** **=** .047* (95% CI: 0.03, 4.58)
eGFR				*B* = −6.35 *P* = .24 (95% CI: −16.9, 4.24)	**eGFR**				*B* = 1.39 *P* = .81 (95% CI: −9.87, 12.66)

Values are regression coefficients (*B*) with 95% CIs. Model 1: unadjusted; Model 2: adjusted for age and sex; Model 3: further adjusted for BMI, smoking, DM, and HT; Model 4: further adjusted for UACR and eGFR. Multivariable analyses were restricted to patients with complete covariates (*n* = 86; one patient had missing UACR data). A *P*-value < .05 was considered statistically significant. *P < .05; **P < .01; ***P < .001. Bold values indicate statistical significance.

aIL-18, active interleukin-18; A/V, arterio-venous; TRPG, tricuspid regurgitant pressure gradient; BMI, body mass index; DM, diabetes mellitus; HT, hypertension; UACR, urinary albumin-to-creatinine ratio; eGFR, estimated glomerular filtration rate.

In an exploratory subset analysis limited to patients meeting HFpEF criteria (LVEF ≥ 50% and HFA-PEFF score ≥ 5), the IL-18 A/V ratio showed a modest, non-significant trend towards higher values compared with non-HFpEF patients (*P* = .058). Associations with *E*/*e*′ and TRPG were not statistically significant in this subset [*E*/*e*′: *r* = 0.30, 95% confidence interval (CI) −0.07 to 0.60, *P* = .108; TRPG: *r* = 0.32, 95% CI −0.08 to 0.63, *P* = .112], although the directions of association were similar to those observed in the overall cohort (see Supplementary data online, *Figure S1*).

To address potential heterogeneity between cases with A/V ratios below and above 1, we performed sensitivity analyses restricted to patients with A/V > 1 (*n* = 47, 54%). In this subset, the correlation with TRPG was attenuated and remained only a trend (*P* = .058, Supplementary data online, *Table S6*). In contrast, the aIL-18 A/V ratio remained consistently and independently associated with *E*/*e*′ across all multivariable models, similar to the overall cohort (see Supplementary data online, *Table S7*). Additional analyses using log-transformed A/V ratios showed concordant directions of association with both *E*/*e*′ and TRPG (see Supplementary data online, *Table S8*), further supporting the robustness of the link between the aIL-18 A/V gradient and LV diastolic function as reflected by *E*/*e*′.

### Associations of interleukin-6 with renal and pulmonary function

Interleukin-6 was significantly associated with UACR (*r* = 0.29, *P* = .006) and per cent vital capacity (%VC; *r* = −0.29, *P* = .013), suggesting potential links to renal and pulmonary impairment (see Supplementary data online, *Figure S2A*, *Table 4*). Interleukin-6 was also inversely correlated with fasting plasma glucose (*r* = −0.28, *P* = .009). In exploratory subgroup analyses, the association between venous IL-6 and UACR was more pronounced in non-hypertensive individuals (sBP < 126 mmHg), whereas the inverse correlation between arterial IL-6 and %VC was evident only in non-diabetic and hypertensive patients (see Supplementary data online, *Figure S2B* and *C*). In contrast to IL-18, the IL-6 A/V ratio was not associated with indices of LV diastolic function (*E*/*e*′ or TRPG; Supplementary data online, *Figure S2D*).

**Table 4:** Regression and correlation analysis of interleukin-6 with clinical parameters

	Arterial (A)			Venous (V)			Arterio-venous (A/V)		
	*r*	*β* (95% CI)	*P*-value	*r*	*β* (95% CI)	*P*-value	*r*	*β* (95% CI)	*P*-value
Age, years	0.11	2.80 (−2.71, 8.30)	.32	**0**.**26**	6.52 (1.28, 11.8)	.015*	−0.15	−1.38 (−3.42, 0.66)	.18
Height, cm^2^	−0.10	−2.83 (−9.42, 3.76)	.40	−0.18	−5.33 (−11.6, 0.97)	.10	0.06	0.64 (−1.82, 3.10)	.61
BW, kg	−0.12	−4.48 (−12.5, 3.58)	.27	−0.19	−6.79 (−14.5, 0.90)	.083	0.08	1.07 (−1.94, 4.08)	.48
BMI, kg/m^2^	−0.09	−0.87 (−3.10, 1.35)	.44	−0.11	−1.10 (−3.23, 1.03)	.31	0.07	0.27 (−0.56, 1.10)	.52
sBP, mmHg	0.21	9.88 (−0.42, 20.2)	.06	0.09	4.12 (−6.07, 14.3)	.42	**0**.**27**	4.81 (1.04, 8.58)	.013*
dBP, mmHg	0.02	0.61 (−7.11, 8.33)	.88	−0.06	−1.97 (−9.53, 5.60)	.61	0.21	2.72 (−0.10, 5.53)	.058
SpO_2_, %	0.07	0.24 (−0.48, 0.97)	.51	−0.02	−0.07 (−0.77, 0.63)	.84	0.09	0.11 (−0.16, 0.38)	.41
FPG, mg/dl	**−0**.**26**	−22.1 (−40.3, −3.91)	.018*	**−0**.**28**	−23.7 (−41.2, −6.18)	.009**	0.06	1.80 (−5.19, 8.79)	.61
HbA1c, %	−0.14	−0.32 (−0.81, 0.17)	.19	−0.17	−0.38 (−0.85, 0.09)	.11	0.07	0.06 (−0.13, 0.24)	.53
UACR, mg/gCr	0.20	0.32 (−0.03, 0.68)	.07	**0**.**29**	0.49 (0.15, 0.84)	.006**	−0.11	−0.06 (−0.20, 0.07)	.32
eGFR, ml/min/1.73 m^2^	0.03	0.01 (−0.06, 0.09)	.77	−0.005	−0.002 (−0.08, 0.07)	.96	0.14	0.02 (−0.01, 0.05)	.21
BNP, pg/ml	0.09	0.14 (−0.19, 0.47)	.39	0.17	0.25 (−0.07, 0.56)	.12	−0.06	−0.03 (−0.16, 0.09)	.59
CTR, %	0.12	2.70 (−2.32, 7.73)	.29	0.17	3.85 (−0.94, 8.64)	.11	−0.05	−0.46 (−2.34, 1.42)	.63
LVEF, %	−0.02	−0.52 (−7.21, 6.17)	.88	0.13	3.97 (−2.55, 10.5)	.23	−0.08	−0.93 (−3.38, 1.53)	.46
LVDd, mm	−0.003	−0.05 (−4.07, 3.98)	.98	−0.03	−0.60 (−4.45, 3.26)	.76	−0.02	−0.11 (−1.59, 1.37)	.88
LVDs, mm	0.01	0.19 (−4.29, 4.67)	.93	−0.04	−0.72 (−5.06, 3.63)	.74	−0.03	−0.24 (−1.89, 1.41)	.77
IVSth, mm	0.07	0.41 (−0.87, 1.69)	.53	0.06	0.37 (−0.88, 1.63)	.56	0.002	0.005 (−0.47, 0.48)	.98
PWth, mm	0.05	0.20 (−0.77, 1.17)	.68	−0.02	−0.09 (−1.04, 0.85)	.84	0.07	0.11 (−0.25, 0.47)	.53
LAD, mm	−0.05	−0.89 (−5.08, 3.29)	.67	−0.05	−0.34 (−4.35, 3.67)	.87	−0.001	−0.01 (−1.55, 1.54)	.99
LAVI, ml/m^2^	0.02	0.97 (−12.9, 14.8)	.89	−0.02	0.22 (−6.53, 19.0)	.33	−0.07	−1.64 (−6.54, 3.27)	.51
*E*/*A*	−0.16	−0.03 (−0.28, 0.22)	.79	−0.12	−0.004 (−0.23, 0.22)	.97	−0.06	−0.02 (−0.11, 0.06)	.58
Septal *e*′, cm/s	0.09	−0.82 (−1.96, 0.33)	.16	0.13	−0.59 (−1.69, 0.51)	.29	−0.04	−0.07 (−0.49, 0.35)	.75
Ave *E*/*e*′	0.07	1.50 (−2.13, 5.13)	.41	0.11	1.98 (−1.49, 5.44)	.26	−0.02	0.11 (−1.21, 1.43)	.87
TRVmax, m/s	−0.05	−0.03 (−0.21, 0.14)	.70	0.13	0.09 (−0.07, 0.25)	.27	−0.19	−0.07 (−0.16, 0.02)	.13
TRPG, mmHg	−0.04	−0.58 (−3.94, 2.79)	.73	0.14	1.78 (−1.31, 4.86)	.26	−0.18	−1.32 (−3.03, 0.40)	.13
HFA-PEFF score	−0.01	−0.04 (−1.07, 0.99)	.94	0.04	0.20 (−0.80, 1.20)	.69	−0.02	−0.03 (−0.41, 0.35)	.89
FEV_1.0%_, %	0.07	1.70 (−4.02, 7.41)	.56	0.14	3.30 (−2.15, 8.74)	.23	−0.18	−2.34 (−5.44, 0.77)	.14
%VC, %	**−0**.**29**	−12.6 (−22.6, −2.72)	.013*	**−0**.**27**	−11.6 (−21.1, −1.99)	.019*	0.06	1.43 (−4.26, 7.12)	.62

Simple regression analysis of arterial (A), venous (V), and arterio-venous (A/V) ratio of IL-6 with clinical parameters.

IL-6, UACR, eGFR, and BNP were log-transformed to approximate a normal distribution. A *P*-value < .05 was considered statistically significant. *P < .05; **P < .01. Bold values indicate statistical significance. Pearson correlation coefficients (*r*), regression coefficients (*β*), 95% CIs, and *P*-value are presented.

IL-6, interleukin-6; BW, body weight; BMI, body mass index; sBP, systolic blood pressure; dBP, diastolic blood pressure; SpO_2_, peripheral capillary oxygen saturation; FPG, fasting plasma glucose; HbA1c, haemoglobin A1c; UACR, urinary albumin-to-creatinine ratio; eGFR, estimated glomerular filtration rate; BNP, B-type natriuretic peptide; CTR, cardiothoracic ratio; LVEF, left ventricular ejection fraction; LVDd, left ventricular diastolic dimension; LVDs, left ventricular systolic dimension; IVSth, interventricular septal thickness; PWth, posterior wall thickness; LAD, left atrial dimension; LAVI, left atrial volume index; TRVmax, maximal tricuspid regurgitant velocity; TRPG, tricuspid regurgitant pressure gradient; HFA-PEFF, Heart Failure Association–Pre-test assessment, Echocardiography and natriuretic peptide, Functional testing, Final aetiology; FEV1.0%, forced expiratory volume in 1 s; %VC, per cent vital capacity.

### Exploratory stratified analysis of combined interleukin-6 and active interleukin-18 levels

Stratified analyses based on venous IL-6 and aIL-18 levels were conducted to examine potential associations with clinical parameters. Patients were categorized into four groups according to median cut-offs: low/low, low/high, high/low, and high/high. No significant differences were detected among the groups in renal, cardiac, pulmonary or composite functional indices (see Supplementary data online, *Figure S3*).

### Profibrotic effects of active interleukin-18 on cardiac fibroblasts

To evaluate the profibrotic effects of aIL-18, HCFs were treated with increasing concentrations of aIL-18 (1–100 ng/ml). Cell proliferation was significantly enhanced, peaking at 10 ng/ml (*P* < .0001) compared with the 0.1% FBS control (see Supplementary data online, *Figure S4A*). Collagen type I alpha 1 expression was also significantly upregulated at 10 ng/ml (*P* = .0494), consistent with the proliferation response. In contrast, α-SMA expression remained unchanged (see Supplementary data online, *Figure S4B* and *C*). These results indicate that aIL-18 promotes fibroblast proliferation and collagen synthesis, supporting its potential contribution to myocardial remodelling.^18,19^

## Discussion

This study investigated whether aIL-18 A/V gradients were associated with indices of diastolic dysfunction in patients undergoing diagnostic cardiac catheterization. The aIL-18 A/V ratio consistently correlated with average *E*/*e*′, a well-established surrogate of elevated LV filling pressure. In simple regression analyses, this correlation appeared more pronounced in subgroups of patients with increased afterload or higher filling pressures. In contrast, absolute arterial or venous concentrations were not related to diastolic indices, suggesting that *trans*-pulmonary gradients may capture information distinct from systemic cytokine levels.

Subgroup analyses indicated that the correlations between the aIL-18 A/V ratio and *E*/*e*′ were more apparent in patients without diabetes or HT, suggesting that IL-18–related activity may be more detectable in the absence of comorbidities. In contrast, in those with these comorbidities, chronic inflammation, metabolic stress, vascular remodelling, and medications may attenuate IL-18–related effects. In the HFpEF-restricted subset, no significant correlations were observed, possibly due to the phenotypic heterogeneity of this population. Nevertheless, sensitivity analyses (restricted to A/V > 1 or log-transformed ratios) yielded concordant results, supporting the consistency of the observed association. These exploratory findings suggest an interplay between comorbidities, therapy, and cytokine activation in HF.

The pulmonary circulation is a plausible site of IL-18 activation, as oxidative stress during oxygenation can trigger inflammasome-mediated endothelial release.^20,21^ Recent studies and experimental models further show that pulmonary endothelial and macrophage–derived cytokines (e.g. IL-1β) aggravate vascular injury and increase cardiac load,^22–24^ while single-cell data implicate macrophage–endothelial interactions in the pathophysiology of HF with diastolic dysfunction.^25^ Elevated pulmonary vascular or LV filling pressures, reflected by increased TRPG or *E*/*e*′, may exacerbate endothelial dysfunction and drive local cytokine activation. The observed venous–arterial increase in aIL-18 may indicate that pulmonary vascular inflammation is a potential site of cytokine activation related to diastolic dysfunction.

A considerable proportion of patients exhibited an A/V ratio <1, indicating higher venous than arterial IL-18 concentrations. Such findings may reflect peripheral tissue release, differential clearance across organs or measurement variability. These cases may represent clinical profiles where local IL-18 activation is less prominent or systemic clearance predominates. Although these findings highlight the complexity of cytokine kinetics, the observed association between a higher A/V ratio and diastolic dysfunction suggests a potential involvement of local IL-18 activation under pressure-stressed conditions.

Cardiomyocytes can also produce IL-18 under mechanical strain and oxidative stress.^12,26^ While such cardiomyocyte-derived IL-18 would initially enter the coronary sinus, subsequent dilution in the right atrium makes it unlikely to explain the observed gradient. Our *in vitro* data confirmed the profibrotic activity of IL-18; however, the gradient may more likely reflect pulmonary release or differences in clearance. Taken together, these observations are consistent with the possibility that IL-18 activation within the cardiopulmonary system could be involved in the inflammatory milieu associated with diastolic dysfunction.

Absolute arterial IL-18 concentrations were not associated with diastolic parameters, whereas the A/V gradient showed consistent associations. Different from arterial concentrations, which mainly reflect systemic influences, the *trans*-pulmonary gradient isolates local activation. This is compatible with the hypothesis that pulmonary inflammation may contribute to myocardial remodelling in part through IL-18. Consistent with this possibility, a previous study in lung injury reported higher arterial than mixed venous IL-6, suggesting net pulmonary release.^27^ Thus, the gradient may serve as an indicator of local activation and may be consistent with the possibility that IL-18 contributes to fibrotic remodelling under certain conditions.

This study has several limitations. It was a cross-sectional, single-centre study with a modest sample size. Blood sampling was not always simultaneous, and invasive haemodynamic parameters such as LVEDP were not obtained. Other inflammatory markers were also not assessed. The study cohort was clinically heterogeneous and not all participants had HF. Many patients underwent catheterization for the evaluation of ischaemic or valvular disease, which likely contributed to the high proportion of preserved EF in this cohort. This clinical background may also have introduced a selection/referral bias towards patients with preserved systolic function. Finally, the *in vitro* experiments employed supra-physiological concentrations of IL-18 to reliably activate IL-18 receptor signalling, limiting direct extrapolation to *in vivo* settings. Additional discussion is provided in the Supplementary data.

## Conclusions

The aIL-18 A/V gradient showed exploratory associations with markers of diastolic dysfunction, particularly in patients with elevated filling pressures. Interleukin-6 was related to renal and pulmonary impairment. As HFpEF-restricted analyses were not significant, these findings should be considered exploratory and require confirmation in larger studies with appropriate phenotype stratification.

## Supplementary Material

xvaf041_Supplementary_Data

## Declarations

### Data Availability

The data that support the findings of this study are available from the corresponding author upon reasonable request.

### Pre-registered Clinical Trial Number

None supplied.
