# Supplementary material for: Arterio-venous gradient of active interleukin-18 is associated with diastolic dysfunction: a cross-sectional study
Source: ESC Heart Fail. 2026 Jan 19;13(1):xvaf041. doi: 10.1093/eschf/xvaf041 (PMC13168766; doi:10.1093/eschf/xvaf041)
Supplement: xvaf041_Supplementary_Data [file xvaf041_supplementary_data.zip › Supplementary_Discussions_Revise_20251001.docx]

**Supplementary Discussions**

Although previous studies have reported elevated IL-18 in obesity ^1^, our study found a modest inverse association with body weight. This discrepancy may reflect differences in population characteristics or measurement methodology, as our assay specifically detected the active form of IL-18 ^2,3^. Experimental evidence that IL-18 promotes energy expenditure and lipid oxidation may also help explain this inverse relationship ^4,5^.

IL-6 was not associated with diastolic dysfunction but correlated with renal (UACR) and pulmonary (%VC) impairment, consistent with its role in organ-specific inflammation ^6,7^. In addition, IL-6 was inversely correlated with fasting plasma glucose, although prior studies have shown inconsistent results, suggesting a context-dependent relationship. The minimal correlation between IL-6 and aIL-18, along with their distinct clinical associations, indicates that inflammatory responses vary by target organ and metabolic state.

**References**

1. Esposito K, Pontillo A, Ciotola M, Di Palo C, Grella E, Nicoletti G, et al. Weight loss reduces interleukin-18 levels in obese women. J Clin Endocrinol Metab 2002;87:3864-3866. doi:10.1210/jcem.87.8.8781.

2. Uchida Y, Nariai Y, Obayashi E, Tajima Y, Koga T, Kawakami A, et al. Generation of antagonistic monoclonal antibodies against the neoepitope of active mouse interleukin (IL)-18 cleaved by inflammatory caspases. Arch Biochem Biophys 2022;727:109322. doi:10.1016/j.abb.2022.109322.

3. Nariai Y, Kamino H, Obayashi E, Kato H, Sakashita G, Sugiura T, et al. Generation and characterization of antagonistic anti-human interleukin (IL)-18 monoclonal antibodies with high affinity: Two types of monoclonal antibodies against full-length IL-18 and the neoepitope of inflammatory caspase-cleaved active IL-18. Arch Biochem Biophys 2019;663:71-82. doi:10.1016/j.abb.2019.01.001.

4. Netea MG, Joosten LA, Lewis E, Jensen DR, Voshol PJ, Kullberg BJ, et al. Deficiency of interleukin-18 in mice leads to hyperphagia, obesity and insulin resistance. Nat Med 2006;12:650-656. doi:10.1038/nm1415.

5. Somm E, Jornayvaz FR. Interleukin-18 in metabolism: From mice physiology to human diseases. Front Endocrinol (Lausanne) 2022;13:971745. doi:10.3389/fendo.2022.971745.

6. Hasegawa M, Fujimoto M, Matsushita T, Hamaguchi Y, Takehara K, Sato S. Serum chemokine and cytokine levels as indicators of disease activity in patients with systemic sclerosis. Clin Rheumatol 2011;30:231-237. doi:10.1007/s10067-010-1610-4.

7. Suzuki D, Miyazaki M, Naka R, Koji T, Yagame M, Jinde K, et al. In situ hybridization of interleukin 6 in diabetic nephropathy. Diabetes 1995;44:1233-1238. doi:10.2337/diab.44.10.1233.
