# Supplementary material for: Arterio-venous gradient of active interleukin-18 is associated with diastolic dysfunction: a cross-sectional study
Source: ESC Heart Fail. 2026 Jan 19;13(1):xvaf041. doi: 10.1093/eschf/xvaf041 (PMC13168766; doi:10.1093/eschf/xvaf041)
Supplement: xvaf041_Supplementary_Data [file xvaf041_supplementary_data.zip › Supplementary_Legends_Revise_20251001.docx]

**Supplementary Legends**

**Figure S1. Subgroup analyses restricted to HFpEF and non-HFpEF patients.**

**(A) Scatter plot showing the correlation between arterial and venous concentrations of aIL-18 in HFpEF patients.**

**(B) Histogram displaying the distribution of aIL-18 A/V ratios (>1 indicates higher arterial than venous concentrations, <1 indicates the opposite).**

**(C) Scatter plot showing the correlation between arterial and venous concentrations of IL-6 in HFpEF patients.**

**(D) Histogram displaying the distribution of IL-6 A/V ratios.**

**(E)** Correlation analyses between arterial and venous IL-6 and aIL-18, as well as their A/V ratios in HFpEF patients.

**(F)** Dot plot comparing aIL-18 A/V ratio between non-HFpEF and HFpEF patients. Plots show individual values with error bars indicating mean ± SD. Statistical comparison was performed using the Mann-Whitney U test.

**(G)** Scatter plots showing correlations between aIL-18 levels (arterial, venous, and A/V ratio) and average E/e′ or TRPG in HFpEF patients.

**(H)** Scatter plots showing correlations between aIL-18 A/V ratio and average E/e′ or TRPG in non-HFpEF patients.

Panels A-C and E-F display *R²* values. *p* < 0.05 was considered statistically significant.

**Figure S2. Association between IL-6 levels and clinical parameters.**

(A) Scatter plots for arterial, venous, and A/V ratio of IL-6 in relation to FPG, ACR, and %VC.

(B) Subgroup analyses stratified by diabetes status (non-DM vs. DM).

(C) Subgroup analyses stratified by systolic blood pressure (sBP <126 vs. ≥126 mmHg).

(D) Scatter plots showing correlations between IL-6 A/V ratio and average E/e′ or TRPG. Each panel displays the *p*-value and *R²*. *p* < 0.05 was considered significant.

**Figure S3.** **Comparison of combined IL-6 and aIL-18 levels with clinical parameters.**

Clinical parameters (UACR, eGFR, EF, IVSth, LAVI, average E/e′, TRPG, %VC) and the HFA-PEFF score stratified by combined high/low IL-6 and aIL-18 levels (LL, LH, HL, HH). Statistical comparisons were performed using one-way ANOVA followed by Tukey’s post-hoc test.

Bar graphs represent mean ± SD, and *p* < 0.05 indicates statistical significance.

**Figure S4.** **Effects of aIL-18 on human cardiac fibroblasts (HCFs).**

(A) BrdU cell proliferation assay in HCFs treated with aIL-18 (1, 10, 100 ng/mL) under serum-free conditions. Bars represent mean ± SD. Statistical significance was assessed by one-way ANOVA with Tukey’s post-hoc test.

(B) Representative immunoblot images of collagen type I alpha 1 chain (COL1A1) and alpha-smooth muscle actin (α-SMA) expression in fibroblasts stimulated with aIL-18 (1, 10, 100 ng/mL). β-actin served as a loading control.

(C) Quantification of COL1A1 and αSMA protein expression normalized to β-actin.

Bars represent mean ± SD. *p* < 0.05 indicates statistical significance.
