# Supplementary figures and images for: Arterio-venous gradient of active interleukin-18 is associated with diastolic dysfunction: a cross-sectional study
Source: ESC Heart Fail. 2026 Jan 19;13(1):xvaf041. doi: 10.1093/eschf/xvaf041 (PMC13168766; doi:10.1093/eschf/xvaf041)

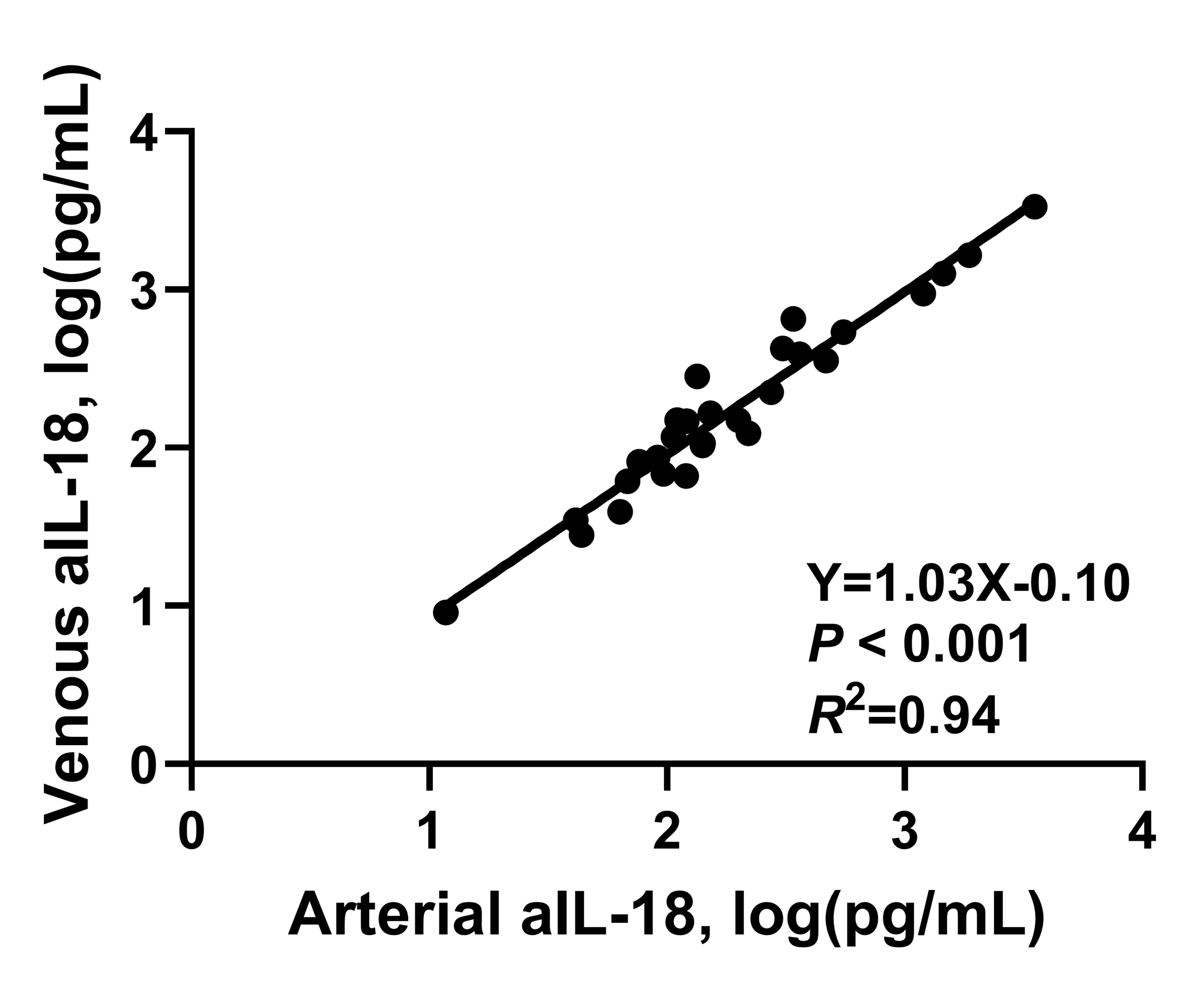

Supplement: xvaf041_Supplementary_Data [file xvaf041_supplementary_data.zip › FigS1A_Revise.tif]

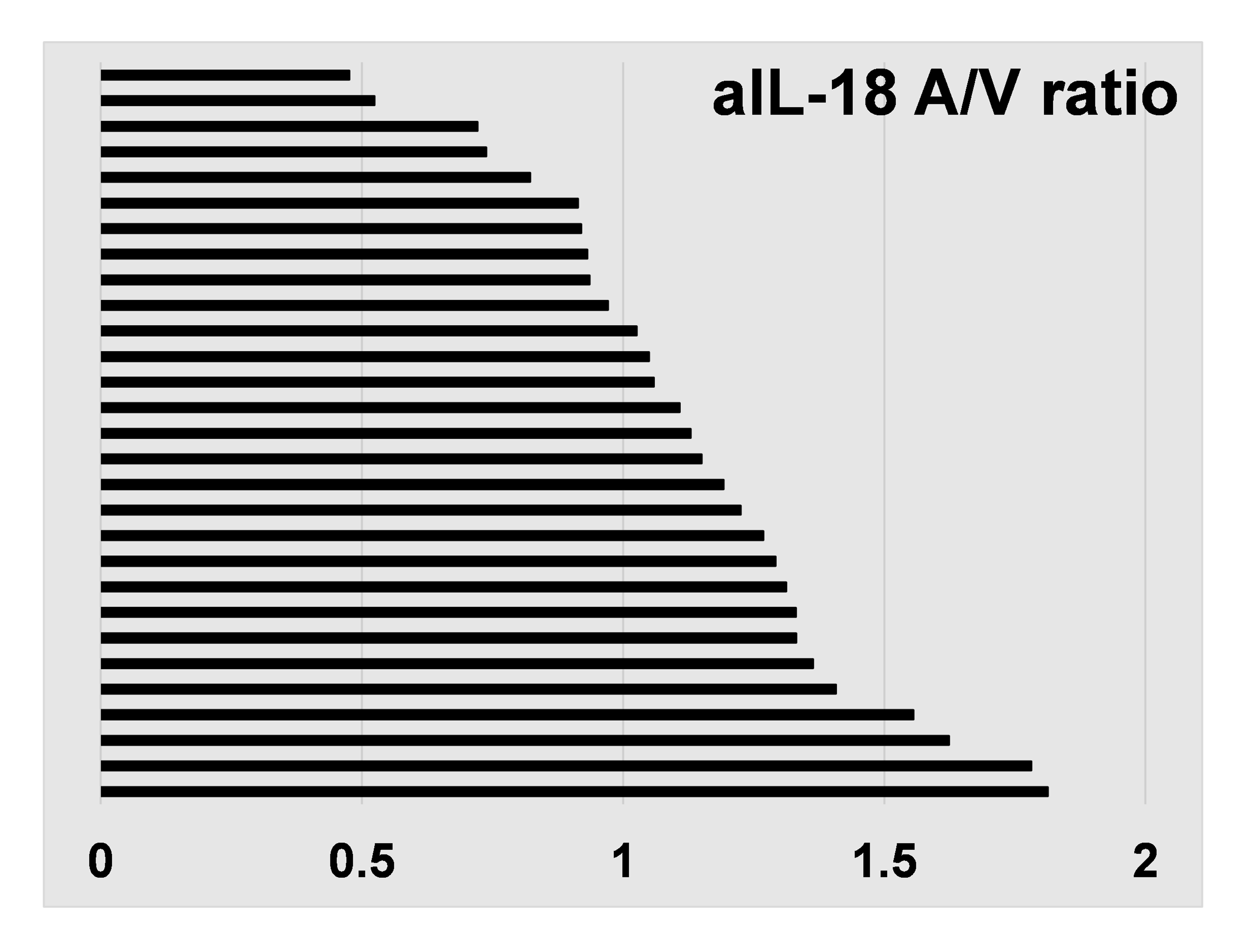

Supplement: xvaf041_Supplementary_Data [file xvaf041_supplementary_data.zip › FigS1B_Revise.tif]

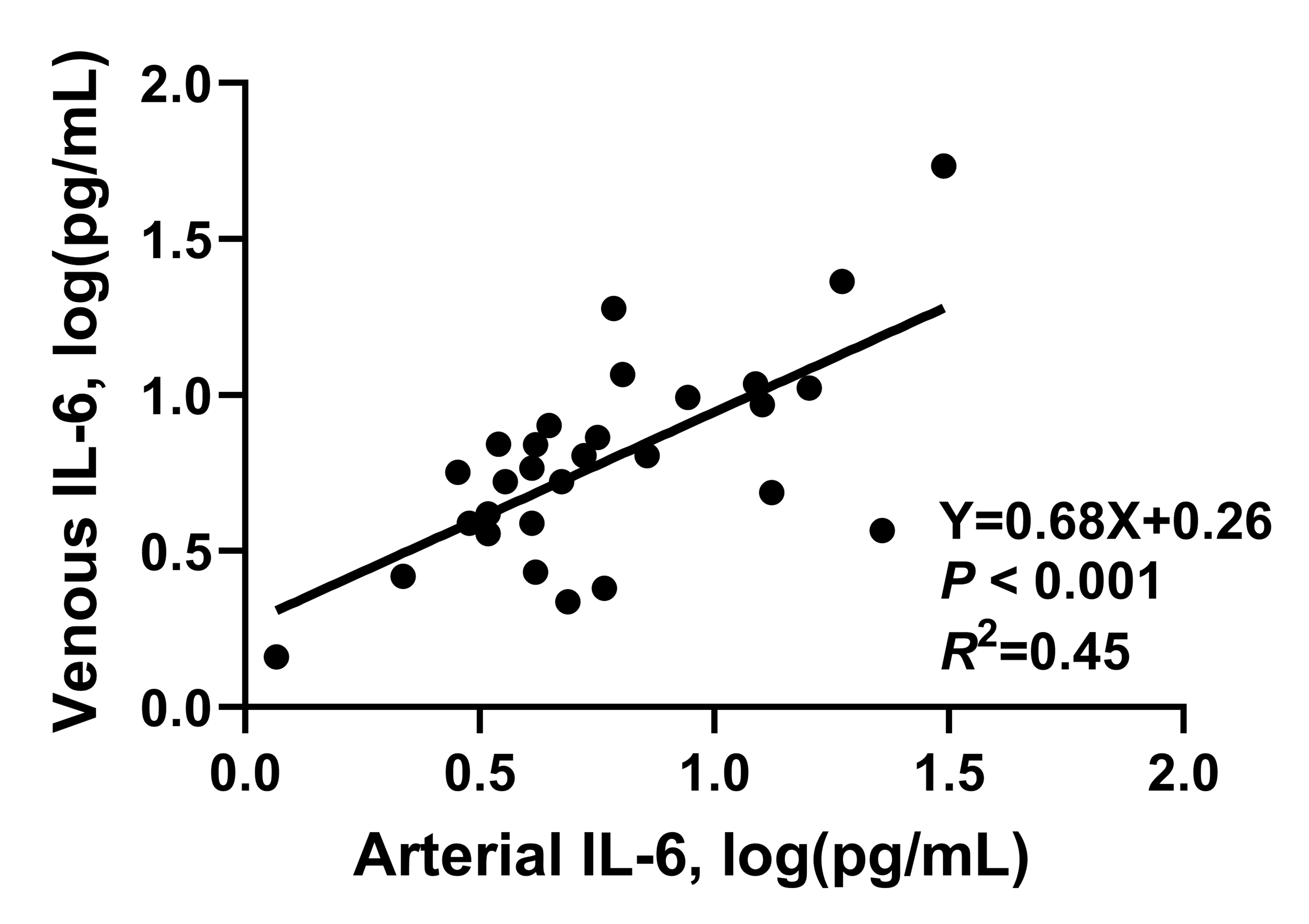

Supplement: xvaf041_Supplementary_Data [file xvaf041_supplementary_data.zip › FigS1C_Revise.tif]

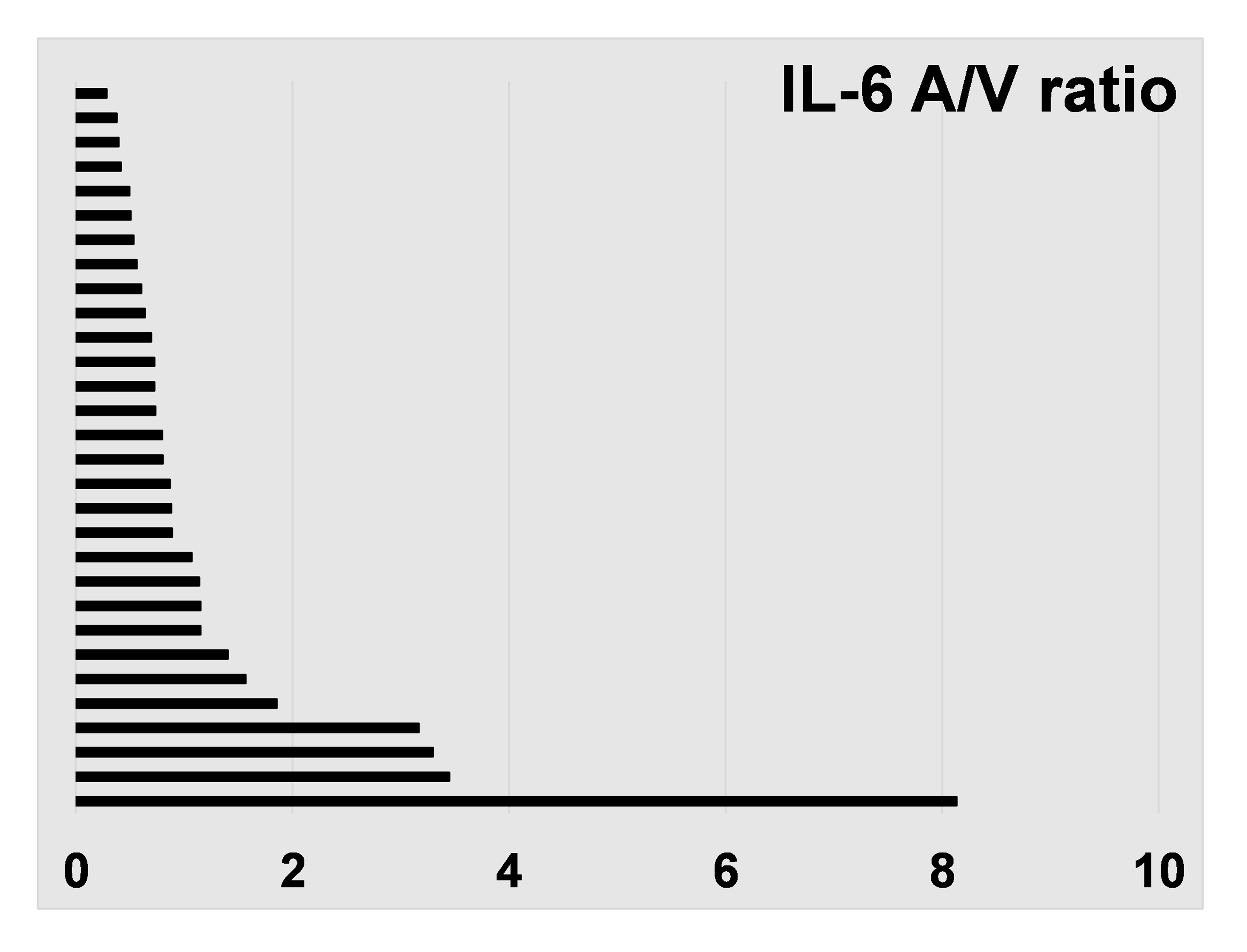

Supplement: xvaf041_Supplementary_Data [file xvaf041_supplementary_data.zip › FigS1D_Revise.tif]

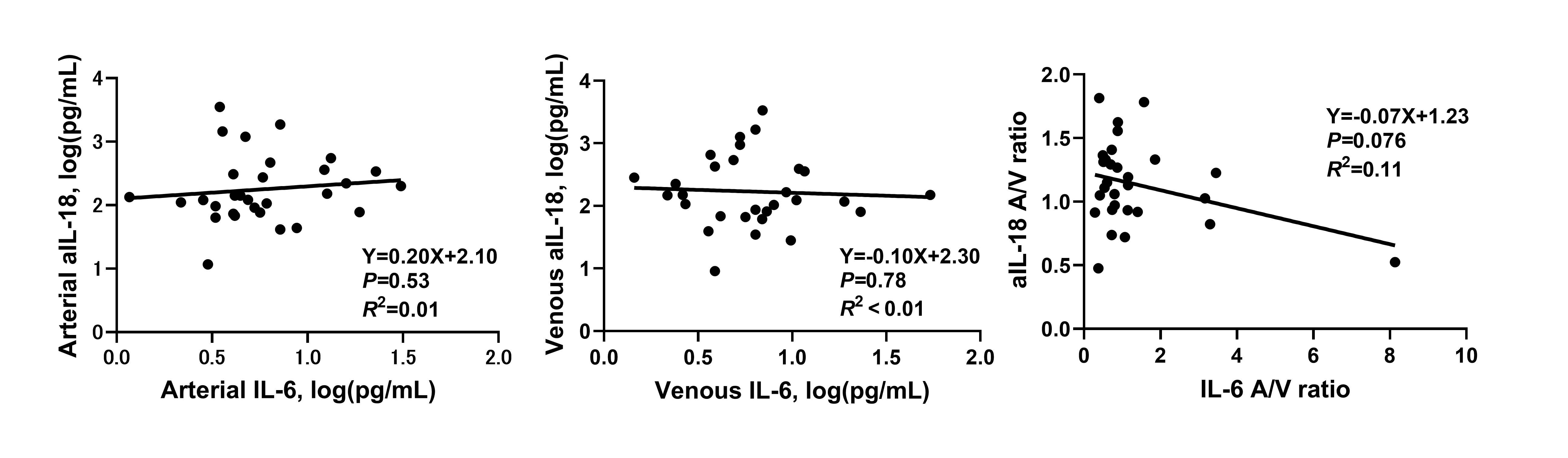

Supplement: xvaf041_Supplementary_Data [file xvaf041_supplementary_data.zip › FigS1E_Revise.tif]

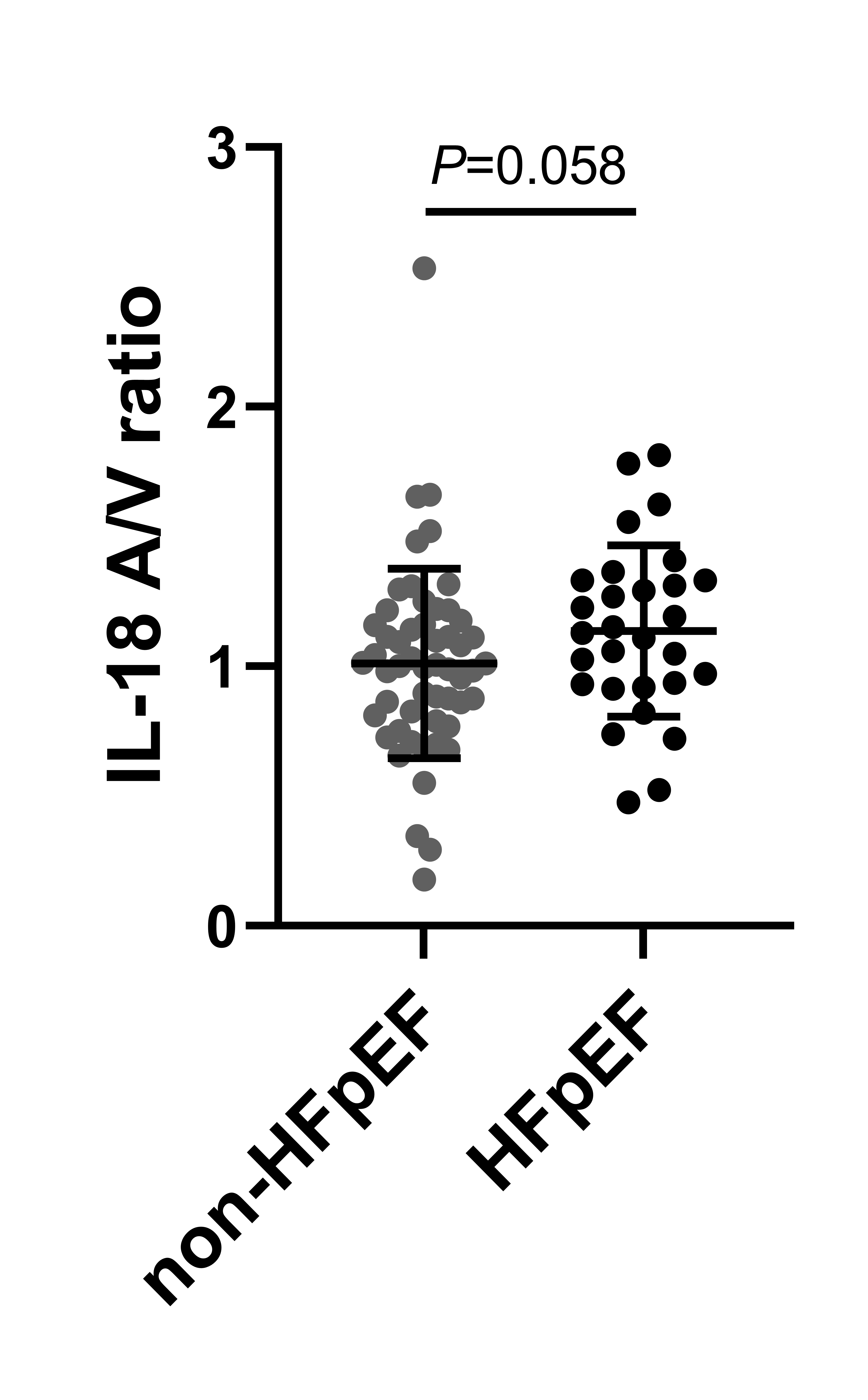

Supplement: xvaf041_Supplementary_Data [file xvaf041_supplementary_data.zip › FigS1F_Revise.tif]

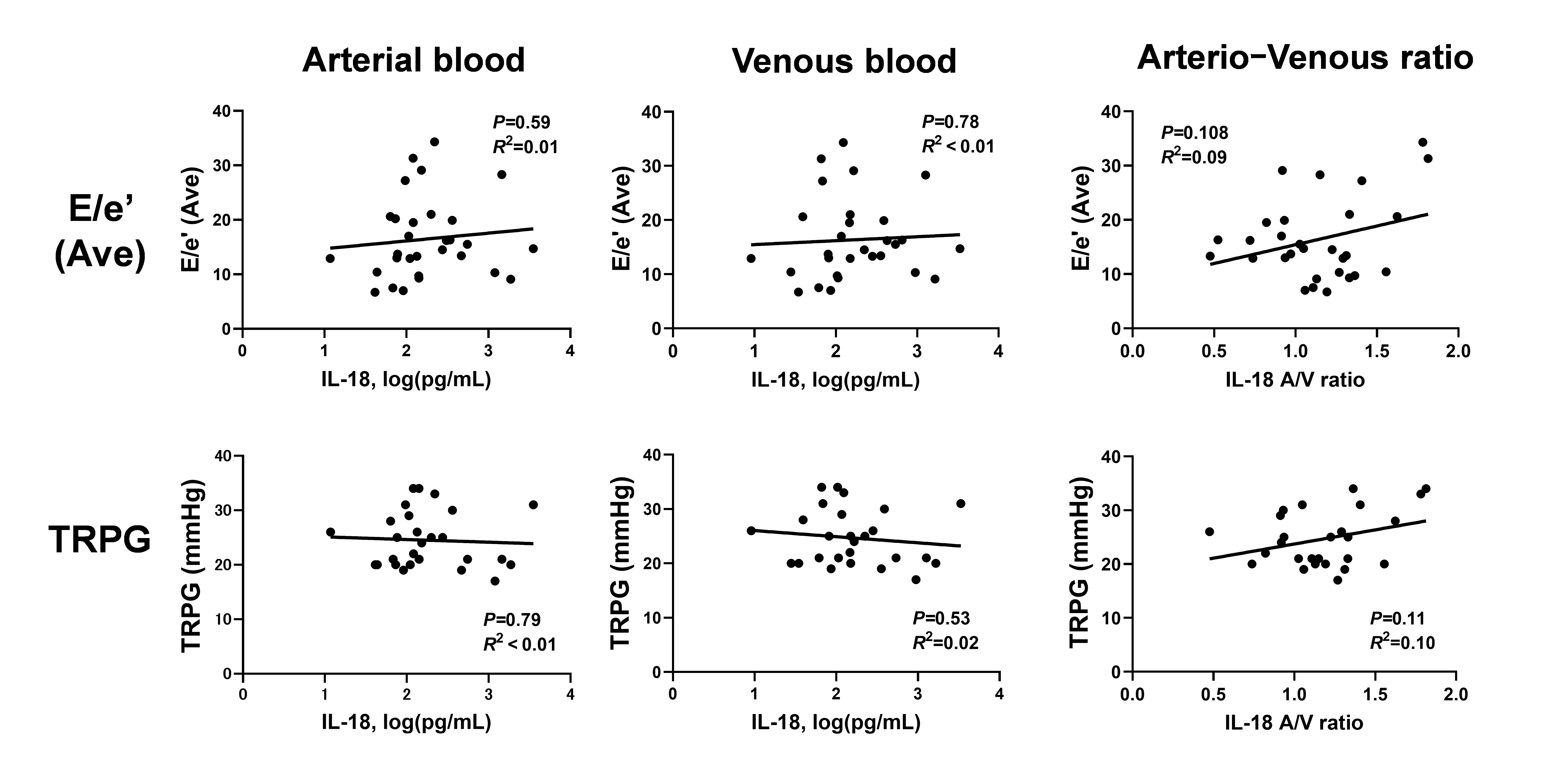

Supplement: xvaf041_Supplementary_Data [file xvaf041_supplementary_data.zip › FigS1G_Revise.tif]

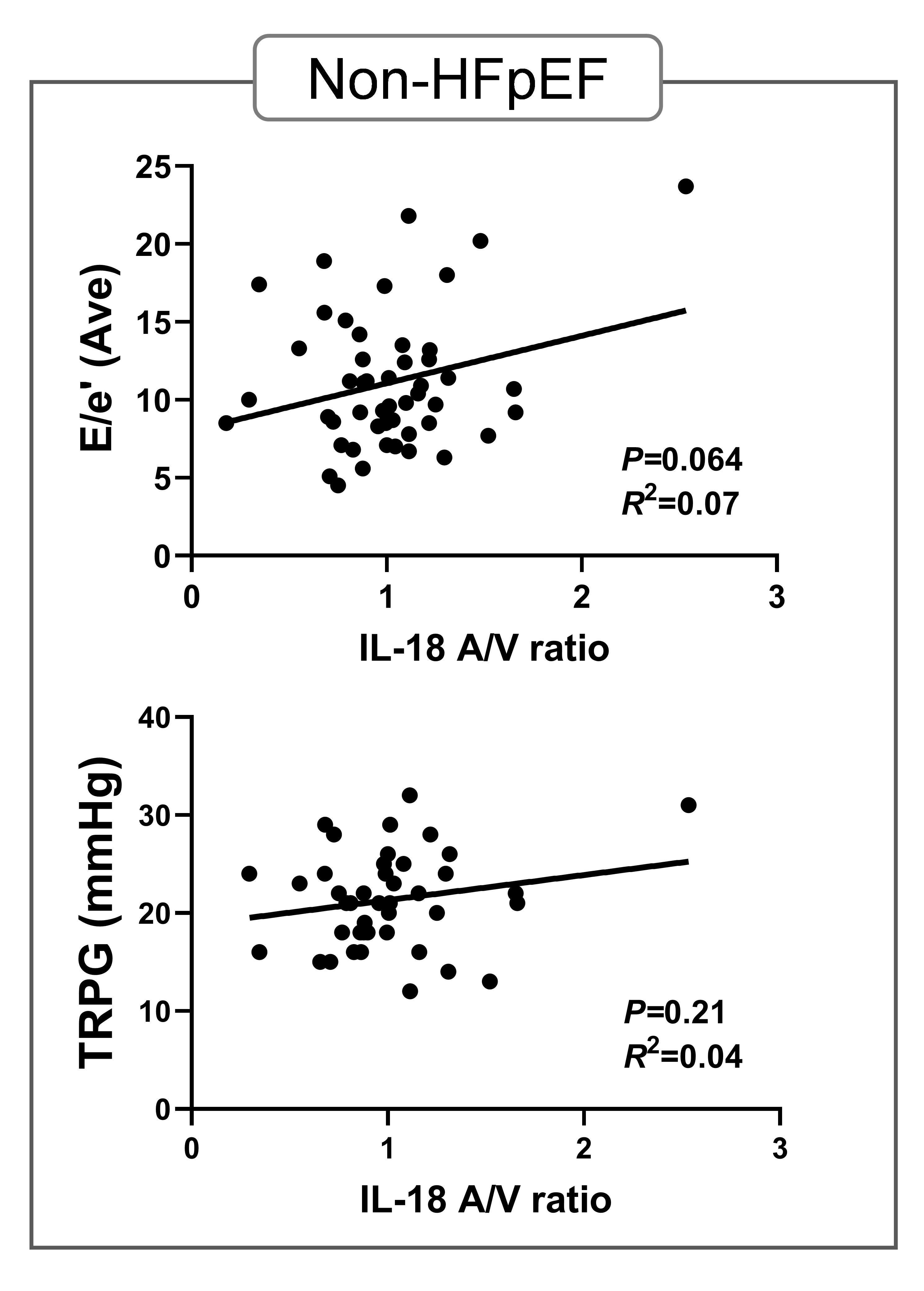

Supplement: xvaf041_Supplementary_Data [file xvaf041_supplementary_data.zip › FigS1H_Revise.tif]

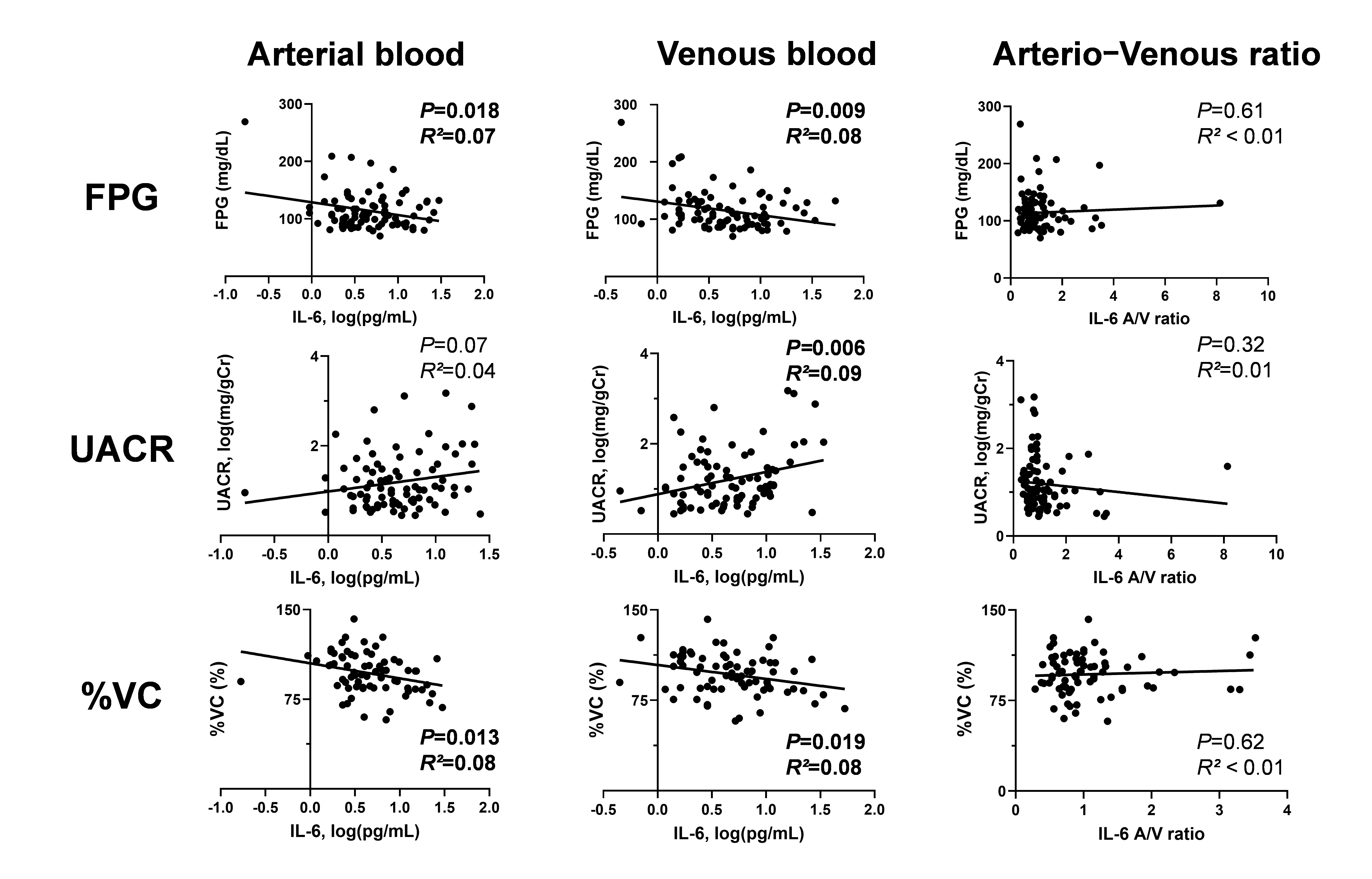

Supplement: xvaf041_Supplementary_Data [file xvaf041_supplementary_data.zip › FigS2A_Revise.tif]

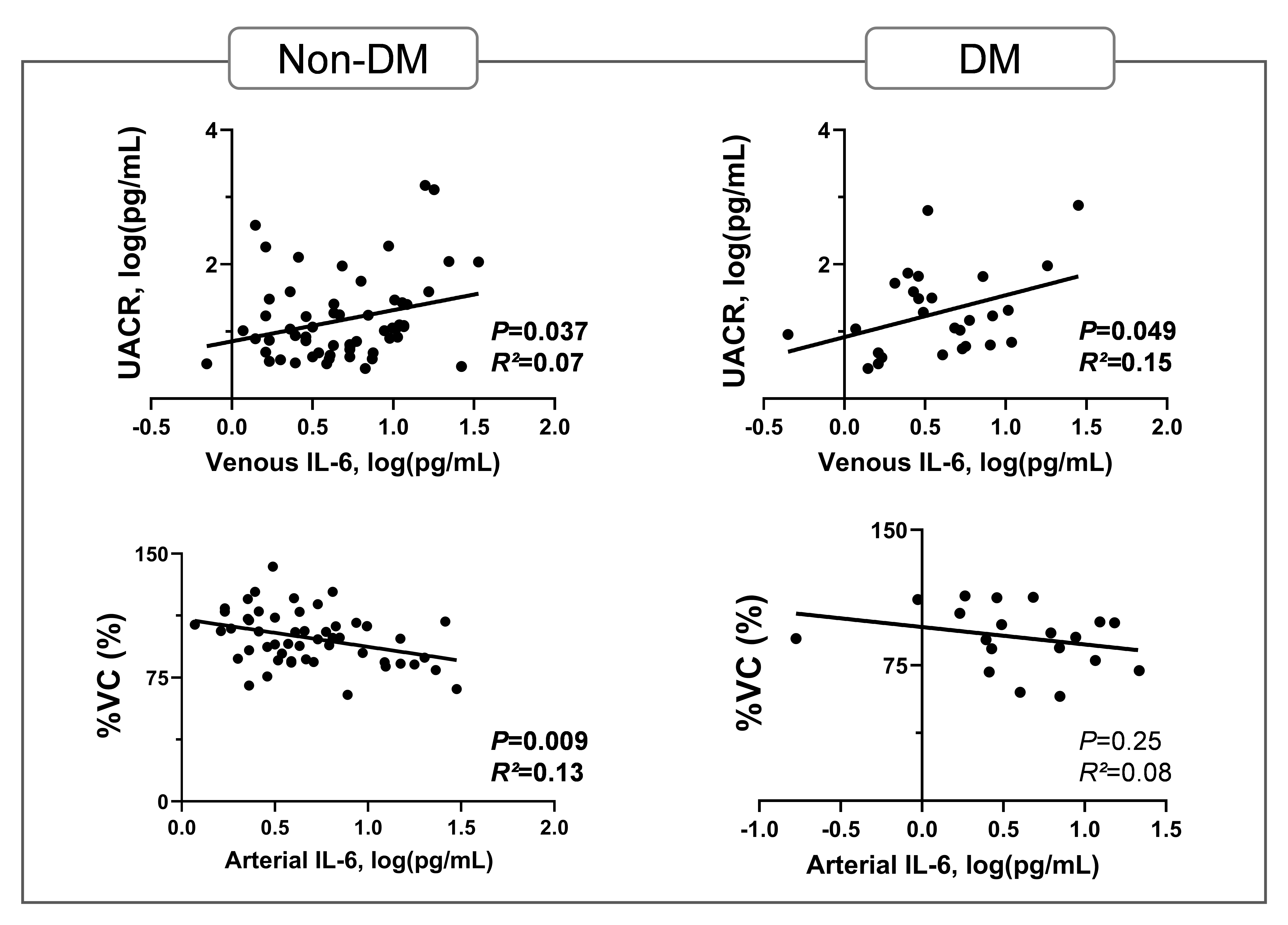

Supplement: xvaf041_Supplementary_Data [file xvaf041_supplementary_data.zip › FigS2B_Revise.tif]

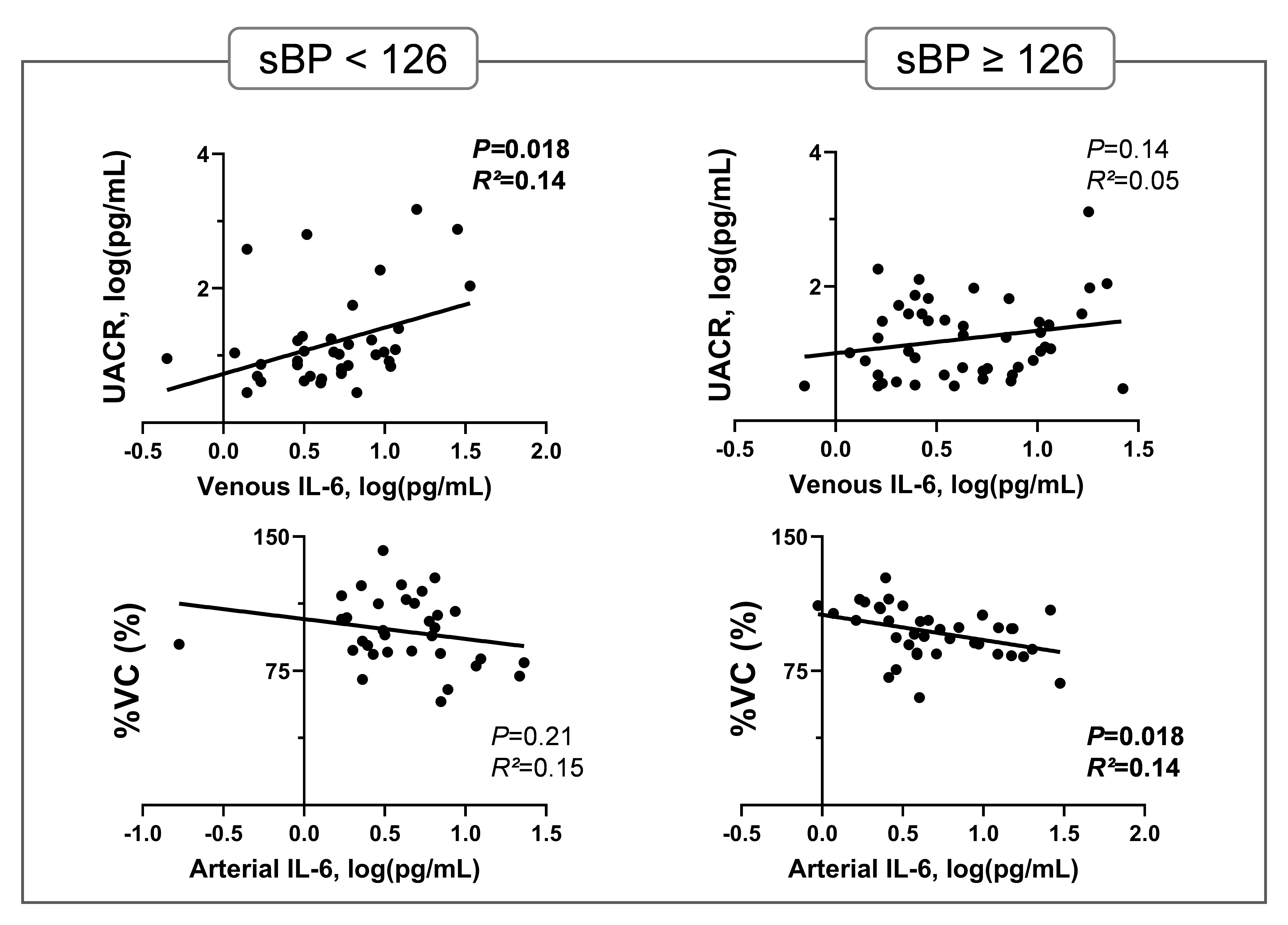

Supplement: xvaf041_Supplementary_Data [file xvaf041_supplementary_data.zip › FigS2C_Revise.tif]

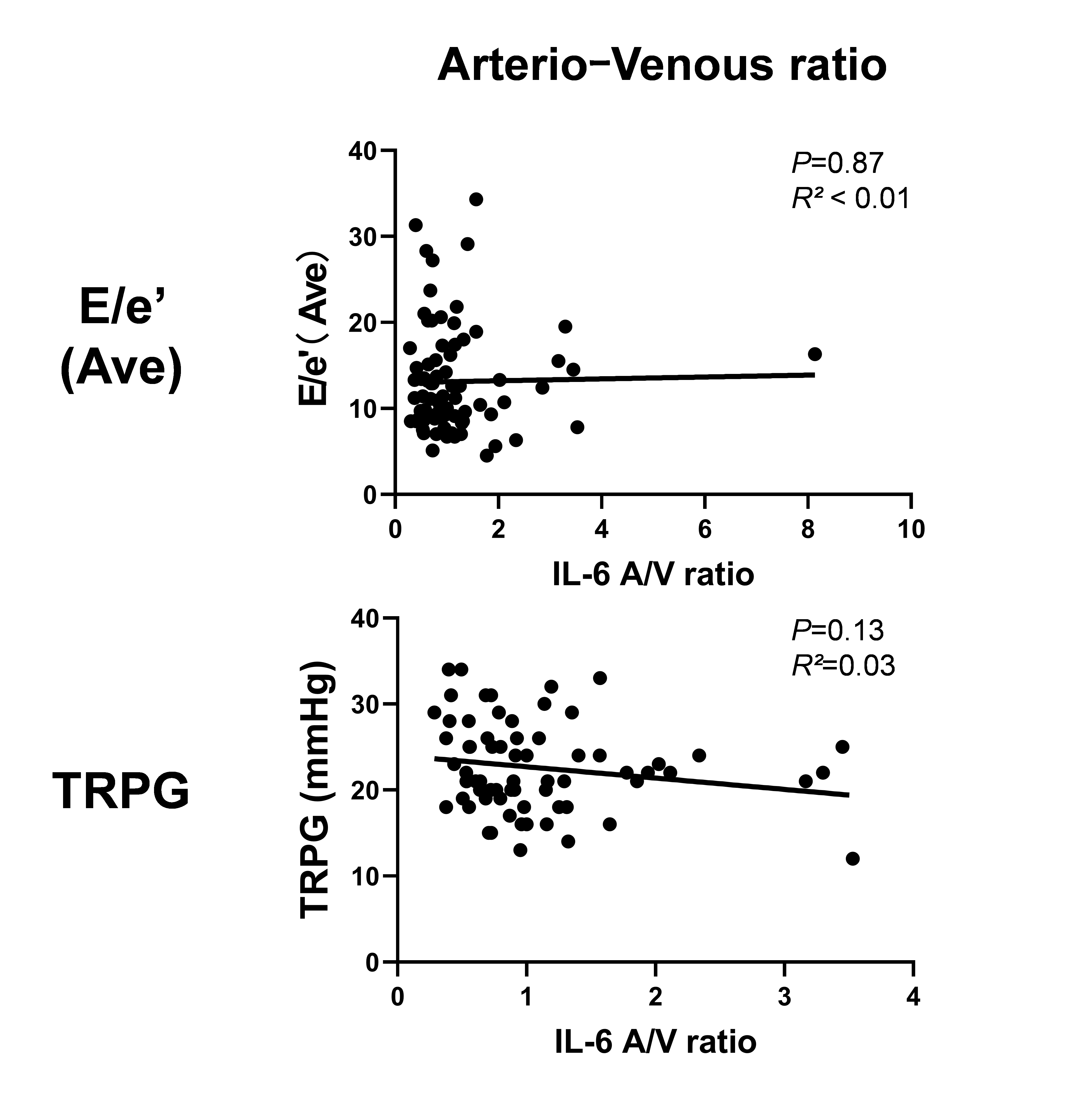

Supplement: xvaf041_Supplementary_Data [file xvaf041_supplementary_data.zip › FigS2D_Revise.tif]

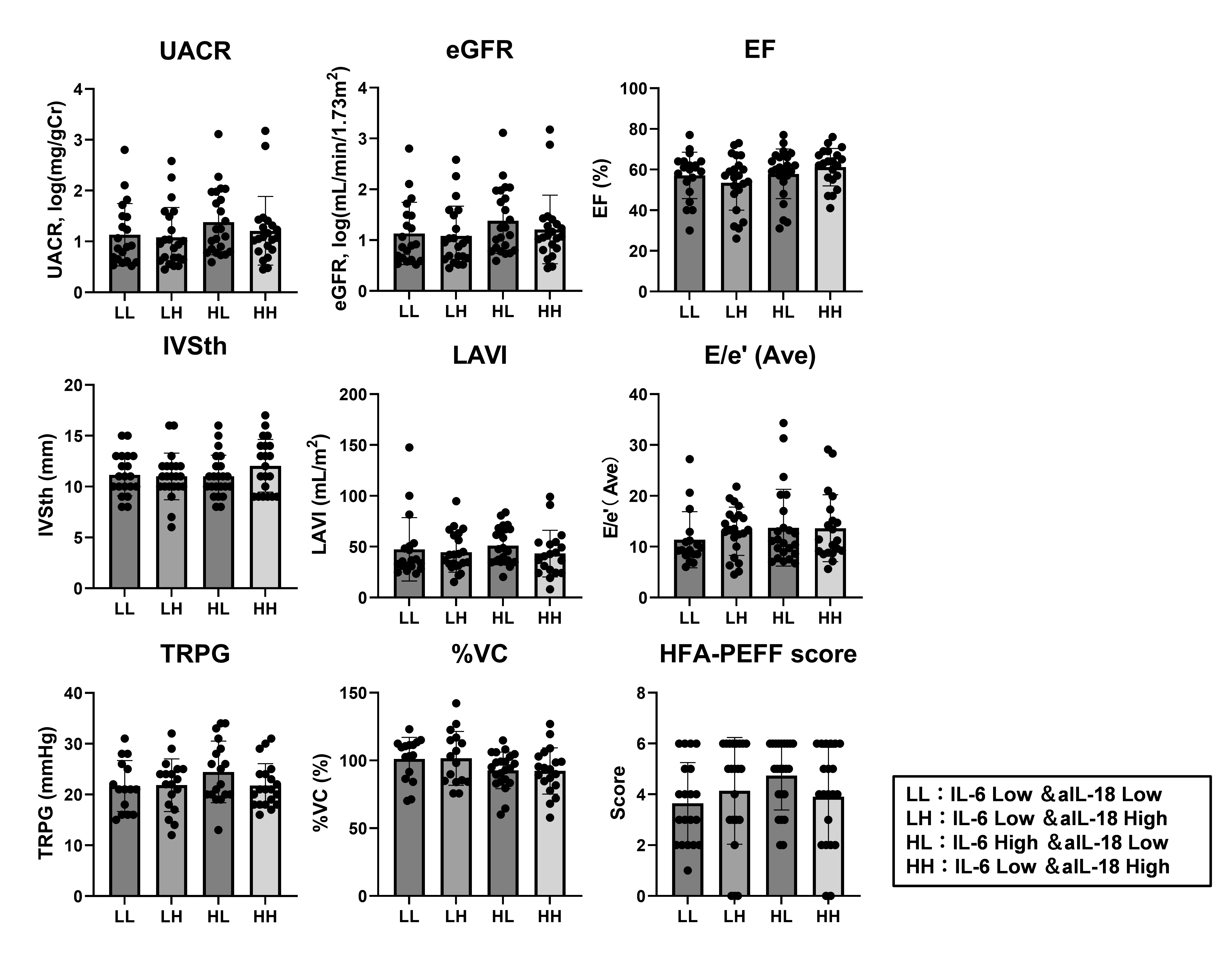

Supplement: xvaf041_Supplementary_Data [file xvaf041_supplementary_data.zip › FigS3_Revise.tif]

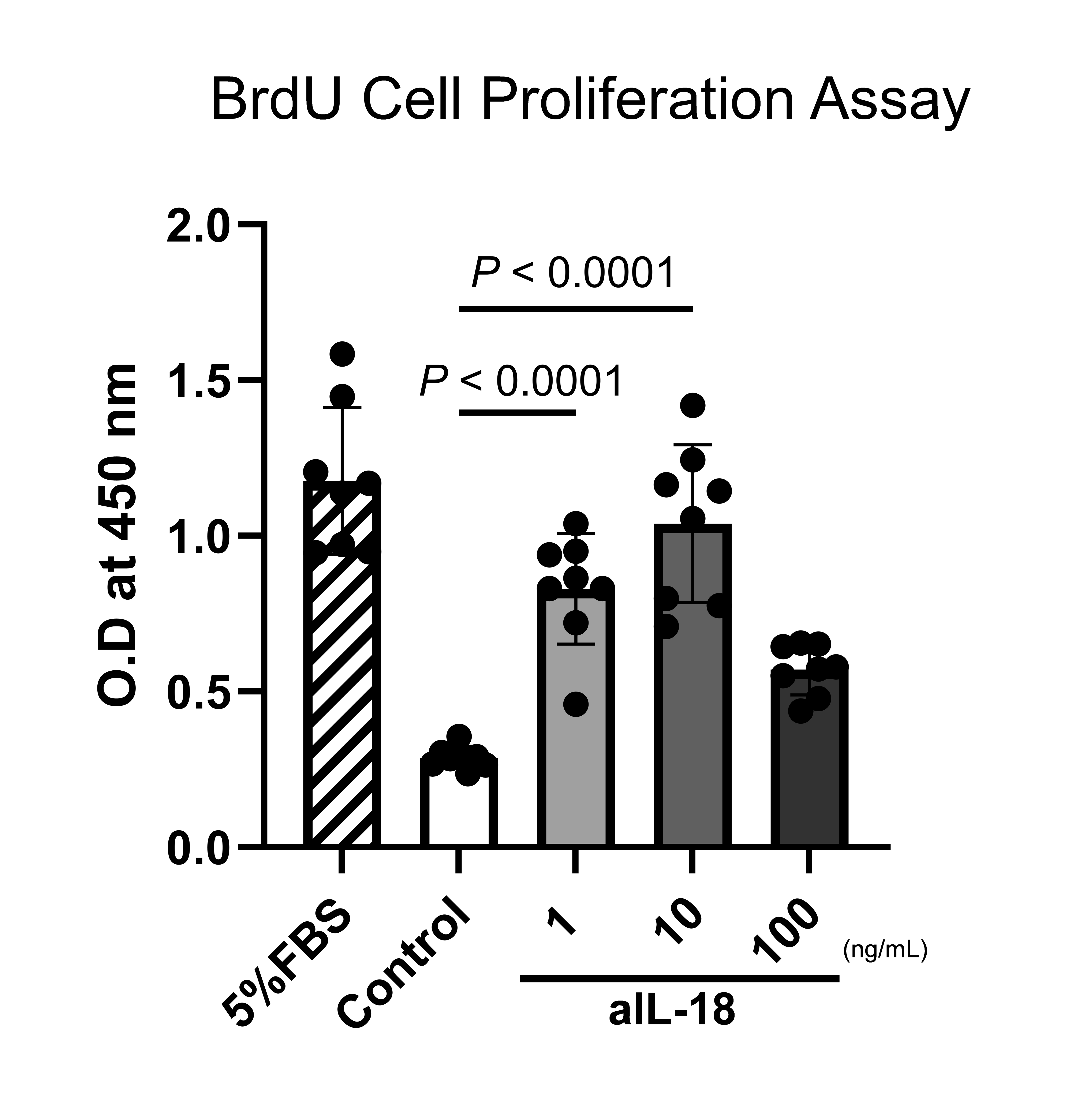

Supplement: xvaf041_Supplementary_Data [file xvaf041_supplementary_data.zip › FigS4A_Revise.tif]

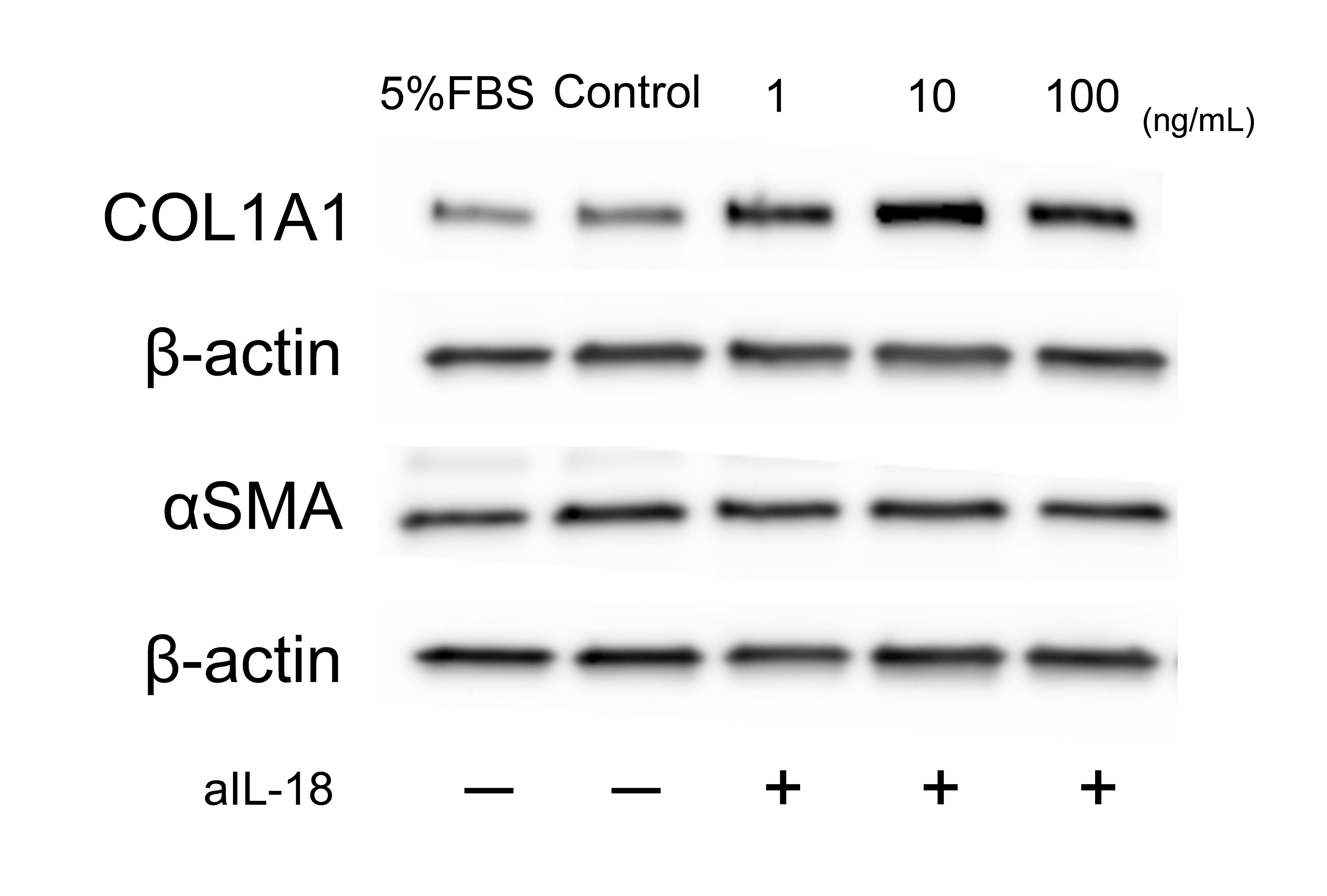

Supplement: xvaf041_Supplementary_Data [file xvaf041_supplementary_data.zip › FigS4B_Revise.tif]

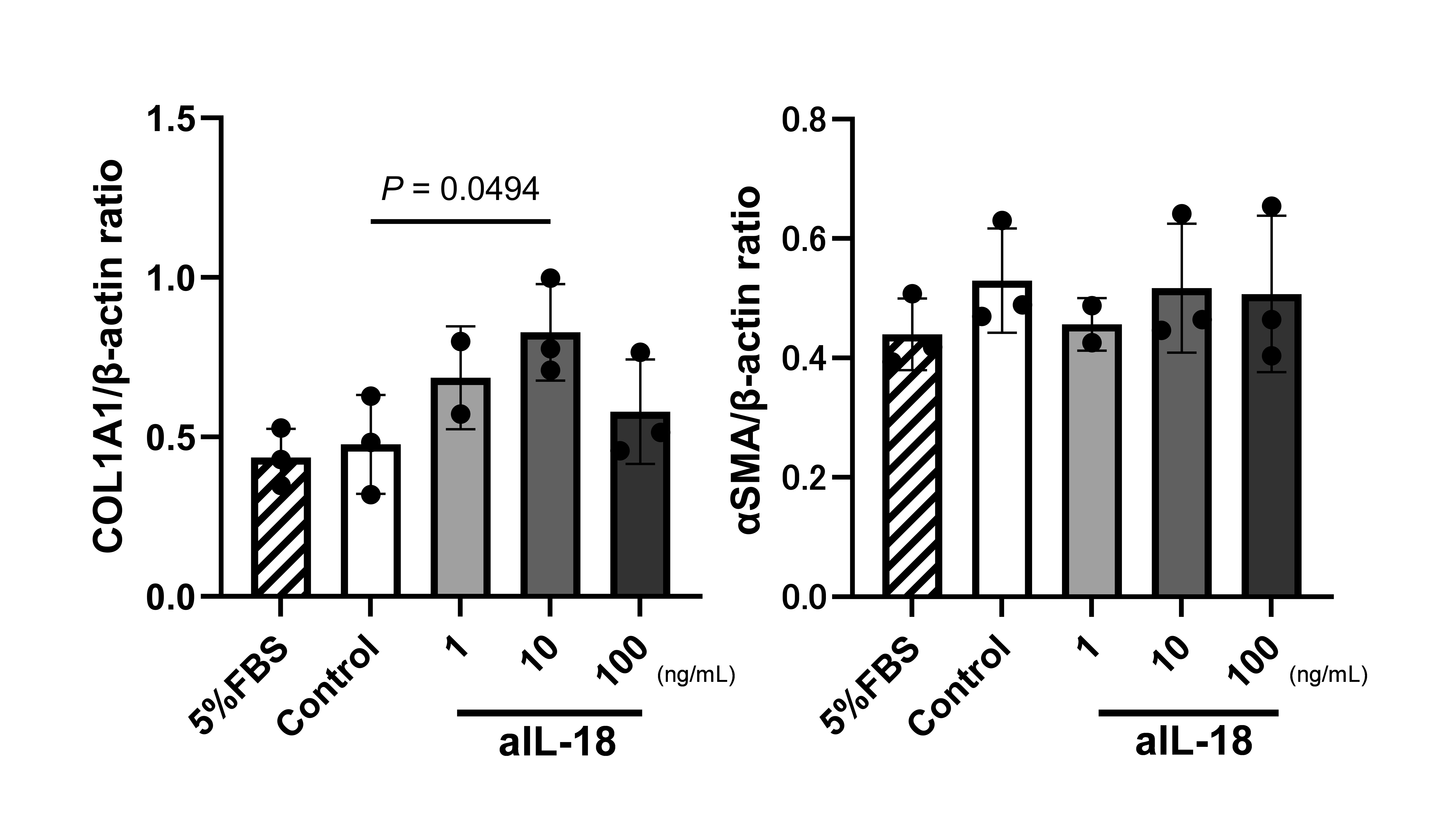

Supplement: xvaf041_Supplementary_Data [file xvaf041_supplementary_data.zip › FigS4C_Revise.tif]

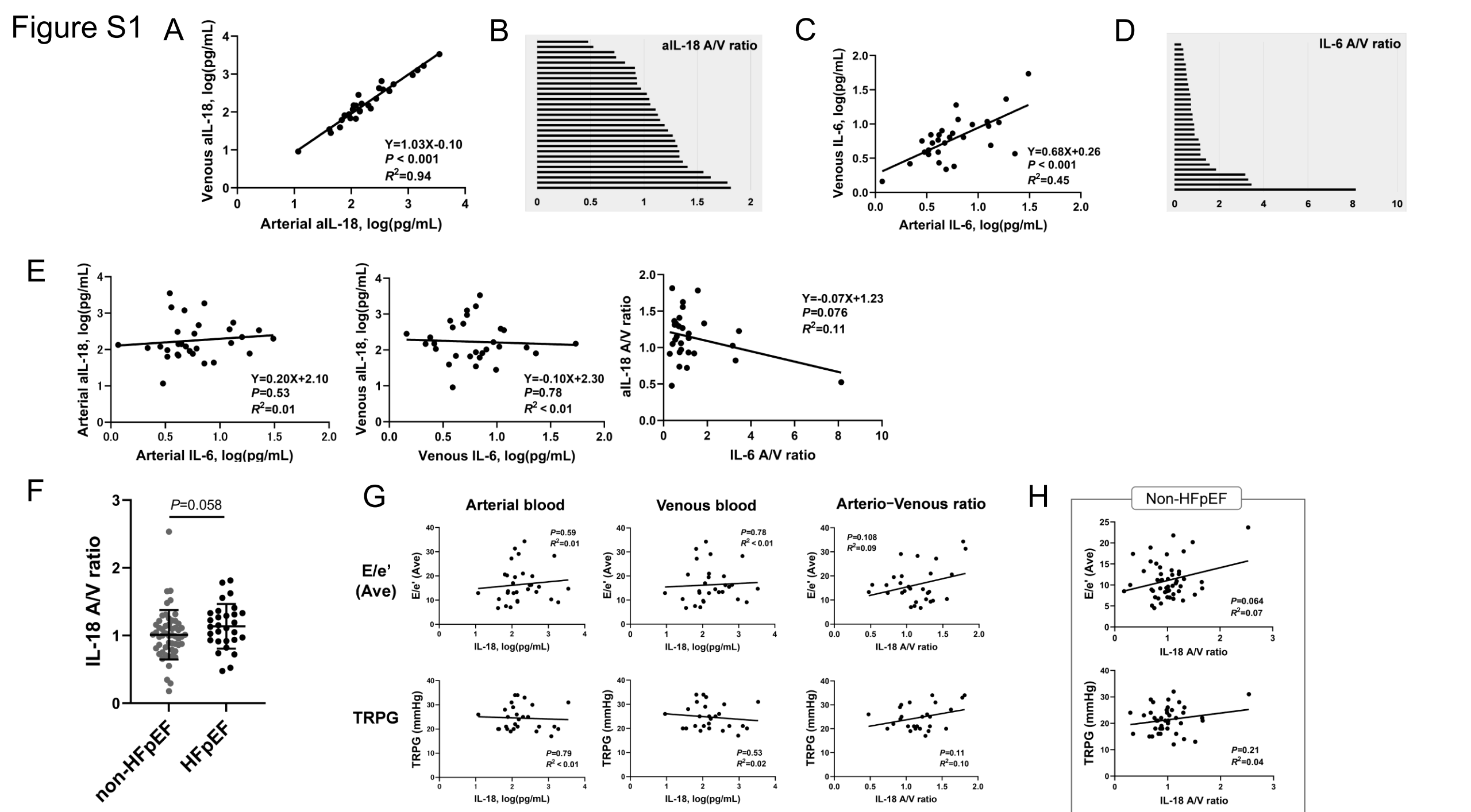

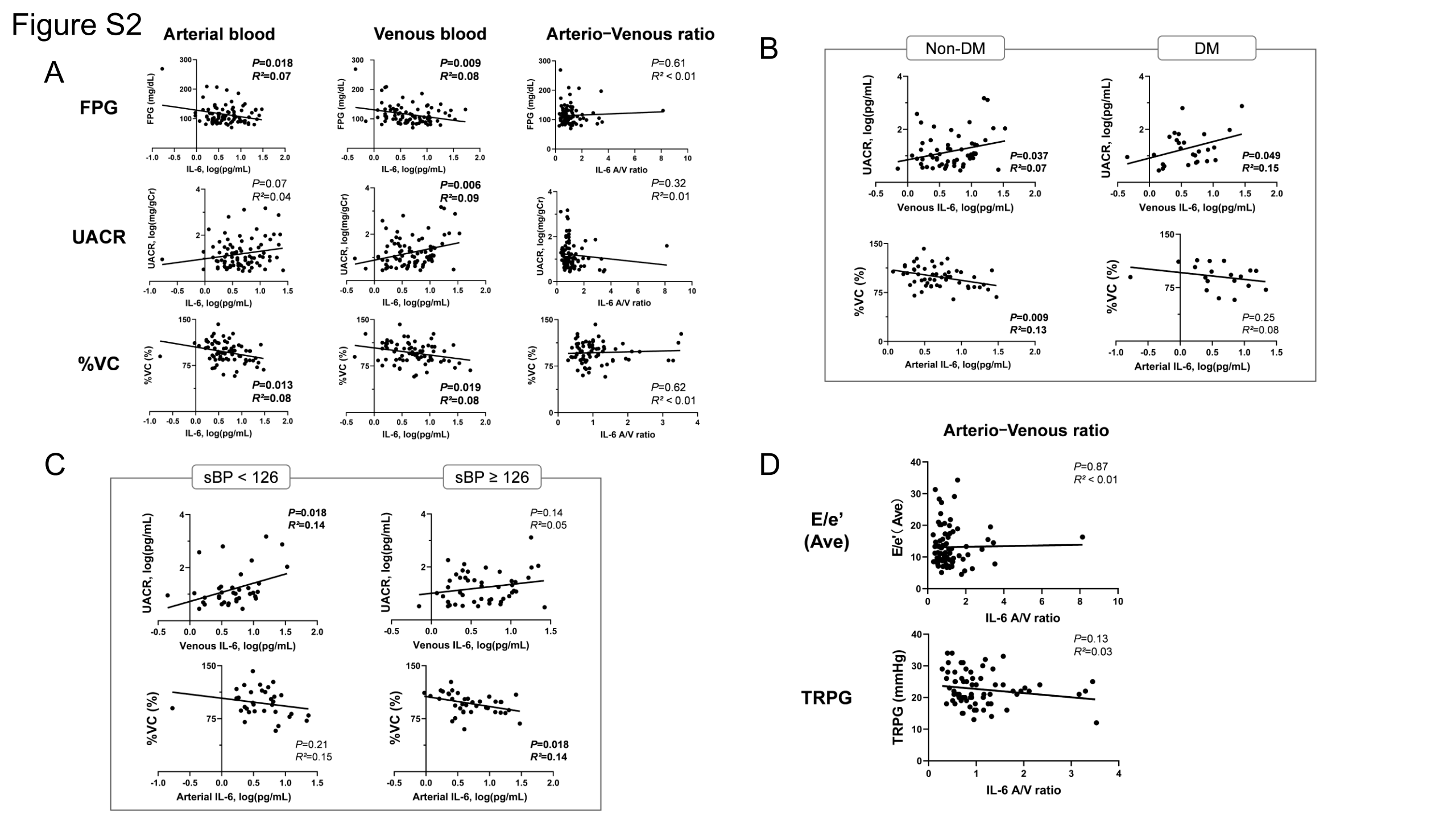

Figure S3

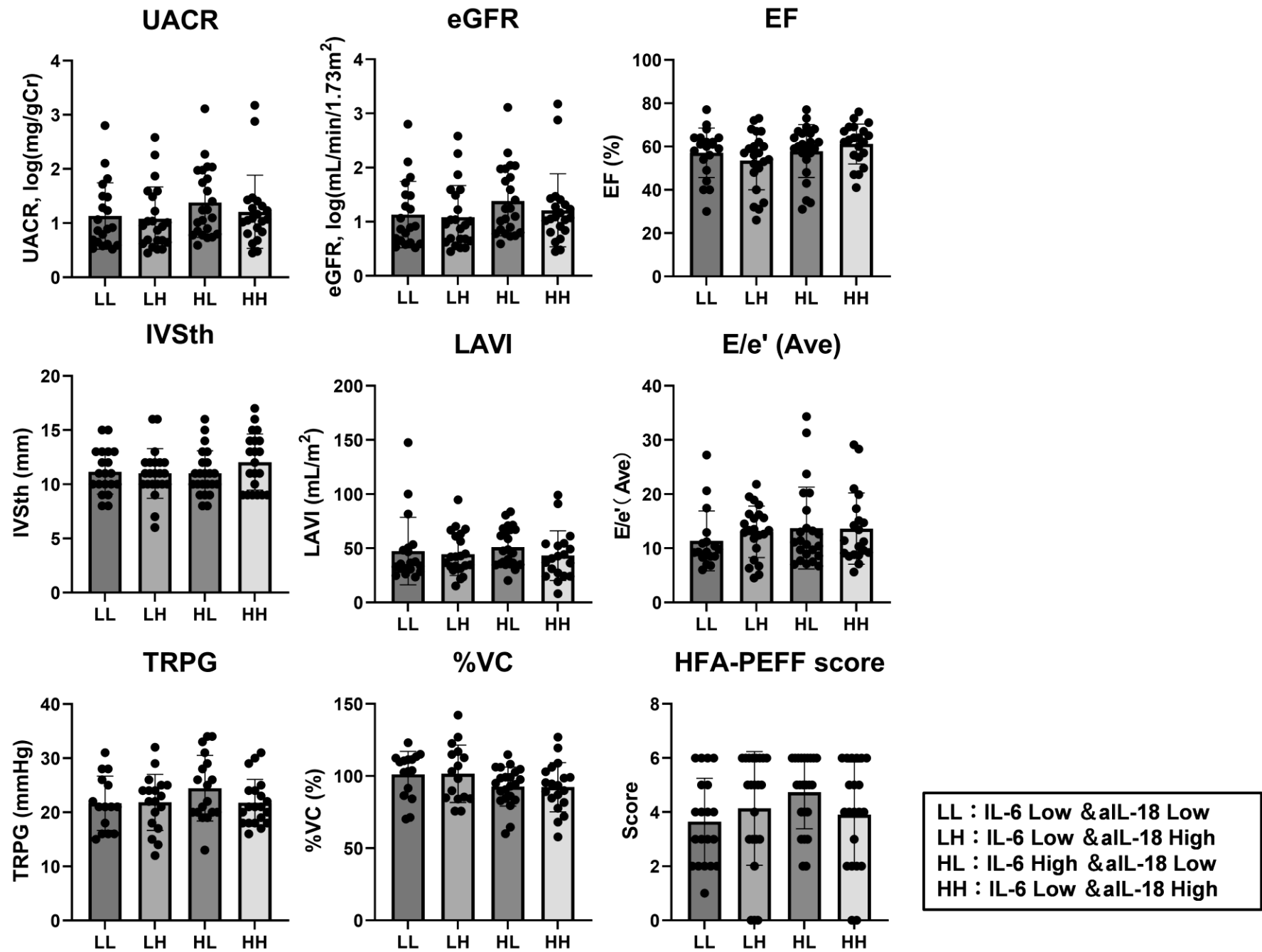

Figure S4

A

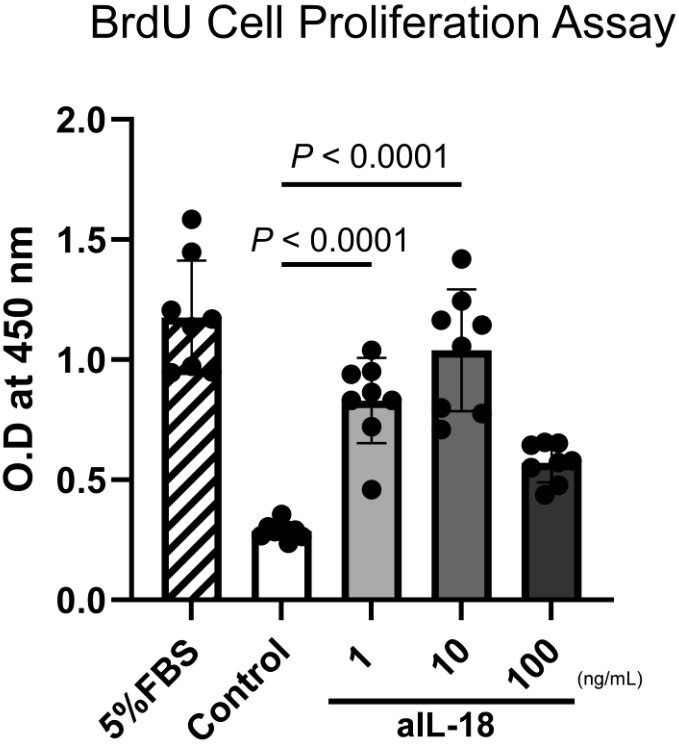

B

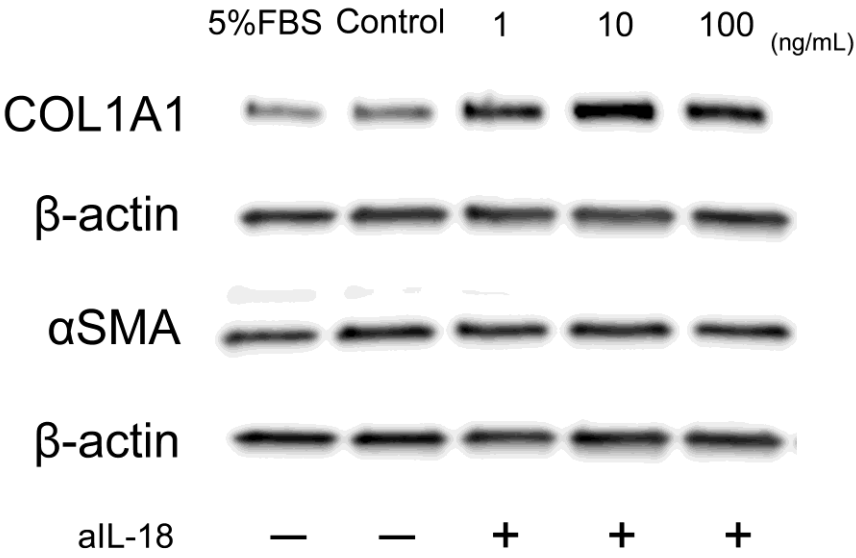

C

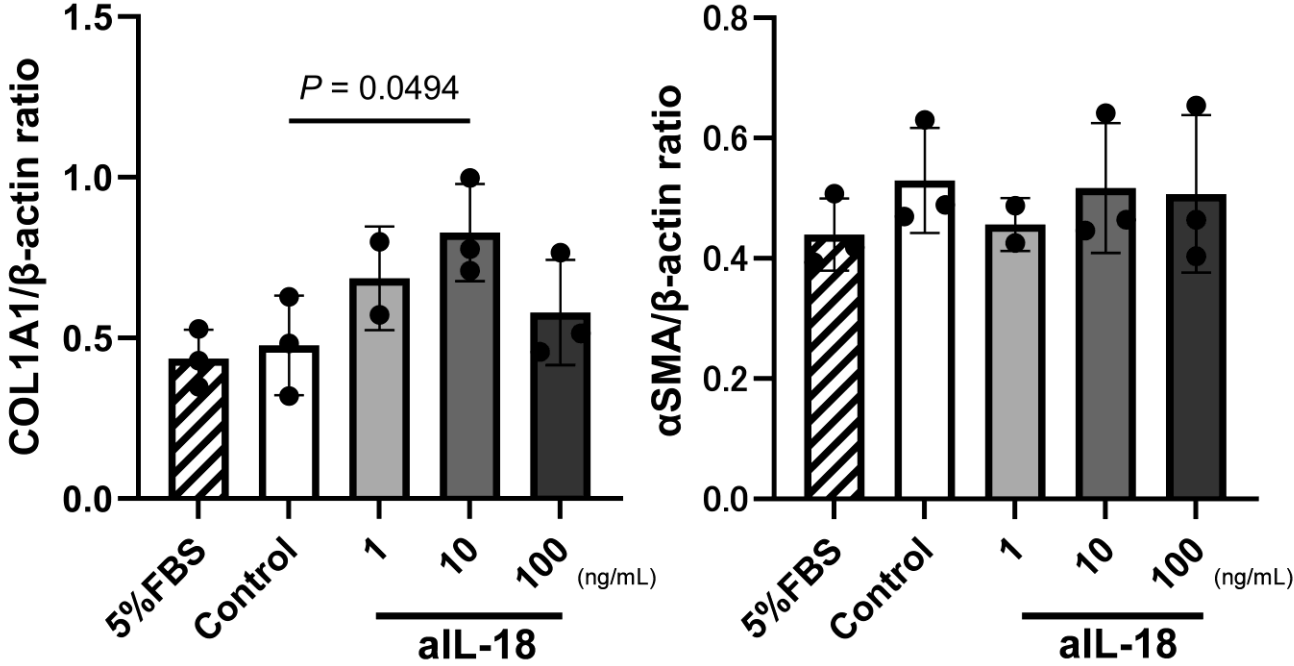

Supplement: xvaf041_Supplementary_Data [file xvaf041_supplementary_data.zip › Supplementary_Figure_Revise_PDF_20251001.pdf]
